# Bioinformatics and Functional Analysis of a New Nuclear Localization Sequence of the Influenza A Virus Nucleoprotein

**DOI:** 10.3390/cells11192957

**Published:** 2022-09-22

**Authors:** Nhan L. T. Nguyen, Nelly Panté

**Affiliations:** Department of Zoology and Life Sciences Institute, University of British Columbia, Vancouver, BC V6T 1Z3, Canada

**Keywords:** influenza A virus, nucleoprotein, nuclear localization sequence, NLS, nuclear import, nucleolar protein 14, NOP14

## Abstract

Influenza viruses deliver their genome into the nucleus of infected cells for replication. This process is mediated by the viral nucleoprotein (NP), which contains two nuclear localization sequences (NLSs): NLS1 at the N-terminus and a recently identified NLS2 (^212^GRKTR^216^). Through mutagenesis and functional studies, we demonstrated that NP must have both NLSs for an efficient nuclear import. As with other NLSs, there may be variations in the basic residues of NLS2 in different strains of the virus, which may affect the nuclear import of the viral genome. Although all NLS2 variants fused to the GFP mediated nuclear import of GFP, bioinformatics showed that 98.8% of reported NP sequences contained either the wild-type sequence ^212^GRKTR^216^ or ^212^GRRTR^216^. Bioinformatics analyses used to study the presence of NLS2 variants in other viral and nuclear proteins resulted in very low hits, with only 0.4% of human nuclear proteins containing putative NLS2. From these, we studied the nucleolar protein 14 (NOP14) and found that NLS2 does not play a role in the nuclear import of this protein but in its nucleolar localization. We also discovered a functional NLS at the C-terminus of NOP14. Our findings indicate that NLS2 is a highly conserved influenza A NP sequence.

## 1. Introduction

The transport of macromolecules into the cell nucleus occurs through nuclear pore complexes (NPCs) via passive diffusion (for molecules up to 5 nm [1,2]) or by a mechanism that requires nuclear localization sequences (NLSs) (reviewed in [3]). Even smaller proteins with sizes below the NPC-diffusion limit require NLSs for a rapid nuclear import or to accumulate to higher levels in the nucleus [4,5,6]. NLSs are usually short stretches of amino acids within proteins recognized by the corresponding soluble nuclear transport receptors of the karyopherin-β superfamily (reviewed in [3,7,8]). The best-characterized NLS, termed the classical NLS (cNLS), consists of either one (monopartite) or two (bipartite) stretches of basic amino acids that are recognized by importin-α [9,10,11]. It has now become apparent that there are many other types of NLSs, such as the proline–tyrosine NLS (PY-NLS) [12], the arginine-rich NLS [13], the arginine/glycine-rich NLS [14], and the lysine-rich NLS [15].

The nuclear import mediated by cNLSs is referred to as the classical nuclear import pathway. In this pathway, importin-α acts as an adaptor between the cNLS and importin-β [11], a karyopherin-β family member. Importin-β docks the NLS–cargo–importin-α/β complex to the NPC and translocates this complex into the nucleus through the NPC [8,16,17,18]. The importin-α structure is an elongated superhelix built from 10 tandem armadillo (ARM) repeats and has two separate NLS binding sites, the major binding site located in the ARM repeats 2–4, and the minor binding site located in the ARM repeats 6–8 [9,19]. The monopartite cNLS binds to the major NLS-binding site, whereas the bipartite cNLS binds simultaneously to both the major and the minor NLS-binding site, with the smaller N-terminal basic cluster bound to the minor binding site and the larger basic cluster bound to the major binding site [9,10]. At the major site, the critical NLS amino acid residues that interact with importin-α are termed P1 to P5, while at the minor site, the critical amino acid residues are termed P1′ to P5′ [9,19].

The influenza A virus is a valuable model for studying nuclear transport because it hijacks the cellular machinery for the nuclear import of its genome, which replicates in the nucleus of the infected cells. The influenza A virus belongs to the *Orthomyxovirus* family, which comprises enveloped viruses with segmented, single-stranded, negative-sense RNA genomes [20]. The influenza A virus genome consists of eight viral RNA (vRNA) segments of variable sizes (reviewed in [21]). Each vRNA segment is packed with the viral RNA polymerase subunits and several copies of the viral nucleoprotein (NP) into viral ribonucleoprotein complexes (vRNPs) [22,23]. NP is a nucleic acid-binding protein, and its primary function is to bind vRNA to encapsidate the virus genome into the RNPs, but it also functions in several steps of the viral infectious cycle [24]. The vRNPs are 15 nm in diameter and 50–150 nm in length, depending on the RNA size [23,25]. Because the vRNP’s size is too large for passing through the NPC by diffusion, influenza vRNPs must display at least one NLS that allows them to hijack the receptor-mediated transport machinery to enter the nucleus through the NPC.

Influenza A NP contains two NLSs (NLS1 and NLS2) that direct the nuclear import of vRNPs and the newly synthesized NP [26,27,28]. The latter must enter the nucleus to bind to the newly synthesized viral RNAs and assemble progeny vRNPs in the nucleus of the infected cells [29]. Based on crystallographic studies, these NLSs are exposed on the surface of the NP [30] and are not involved in direct interactions with the vRNA [31,32,33]. NLS1 is an unconventional NLS located at the N-terminus of the NP (^1^MASQGTKRSYEQM^13^) that binds to the minor NLS-binding site of importin-α [34]. This is not a unique feature of NLS1 because other non-classical NLSs also bind exclusively to the minor NLS-binding site of importin-α [35,36,37,38,39]. NLS2 was hypothesized to be a classical bipartite NLS with two basic amino acid clusters (^198^KR^199^ and ^213^RKTR^216^) separated by a 13 amino acid linker region (^198^KR-GINDRNFWRGENG-RKTR^216^) [40]. However, the crystal structure of a peptide spanning the hypothetical 19-residues long bipartite NLS2 bound to importin-α revealed that only the second cluster of basic residues (^212^GRKTR^216^) interacts with importin-α, both at its major and minor binding site, but mainly at the major site [28]. Similarly, crystallographic studies have also shown that other putative cNLSs previously reported to be bipartite are, in fact, monopartite NLSs with only one basic cluster interacting with importin-α [41].

Based on the structural analysis of NLS2 bound to importin-α, NLS2 was defined as an atypical monopartite NLS with the arginine at position 213 (R213) occupying position P2 of the major NLS-binding pocket of importin-α [28]. Further in vitro binding assays of importin-α and NP with mutations in NLS1 or NLS2 indicated that both NLS1 and NLS2 contribute to the binding of the NP to importin-α [28]. Other studies using an antibody inhibition assay and peptide competition experiments showed that NLS2 alone could mediate the nuclear import of purified vRNPs, suggesting that NLS2 also plays a critical role in the nuclear import of the viral genome during infection [42]. In agreement with this suggestion, competition assays of NLS2 in infected cells revealed that NLS2 is indispensable for viral infection [28]. Based on these studies, the current model for the nuclear import of the influenza NP is that NLS2 functions in synergy with NLS1 as a bipartite NLS that forms only in the tertiary (or quaternary) structure of the NP [28]; NLS2 interacts with the major and NLS1 with the minor NLS-binding pockets of importin-α [28].

During infection, the newly synthesized NPs are targeted to the nucleolus, which is the site for the formation of functional vRNP [43]. In addition to the function in the nuclear entry of the NP, NLS2 also plays a role in the nucleolar localization of the NP. Alanine substitutions of the basic residues of NLS2 impact the nucleolar localization of the NP [43,44]. Thus, NLS2 has been proposed to be a nucleolar localization signal (NoLS) of the NP [43,44]. However, this sequence seems to work as a NoLS only in the context of NP because when it is fused to GFP, GFP does not localize in the nucleolus [28].

As NLS2 is a recently identified NLS, in this study, we investigate the NLS2 contribution to the nuclear import of the NP and confirm that there is synergy between NLS1 and NLS2 because mutations of either NLS1 or NLS2 significantly reduce the nuclear accumulation of NP. We also performed bioinformatics analyses to study whether other natural variants (with different combinations of K and R at different positions) of NLS2 exist in the NP from other influenza virus strains and/or types. We found that two predominant NLS2 sequences, ^212^GRKTR^216^ and ^212^GR**R**TR^216^, are present in the NP of different influenza A virus strains but not in the NP from influenza B or C viruses. Functional studies showed that all NLS2 variants mediated the nuclear import of chimeric proteins to a similar extent. However, as previously reported [28], the ^212^GR**R**TR^216^ sequence yielded a significantly reduced nuclear import. We also extended our bioinformatics analyses to other viral and cellular proteins known to function in the nucleus of different organisms and found that the sequence GRKTR and some of its variants are found in the primary structure of very few of these proteins. We chose to study the role of NLS2 and other putative NLSs in the nucleolar protein 14 (NOP14), one of the protein hits of our analyses. We found that NLS2 does not play a role in the nuclear import of this protein but in its nucleolar localization. In addition, we identified a novel NLS at the C-terminus of NOP14.

## 2. Materials and Methods

### 2.1. Bioinformatics

Protein sequence alignments were carried out using the BLASTP [45] web interface of the National Center for Biotechnology Information (NCBI), available at https://blast.ncbi.nlm.nih.gov (accessed on 20 August 2022). The sequence GRKTR and its variants were compared to sequences of the NP from influenza A, B, C, and D viruses and proteins from DNA virus families available in the non-redundant database. For the latter, only the hits from proteins known to localize in the nucleus of infected cells, as reported in the literature, were considered real hits. Similarly, to search for the presence of the GRKTR sequence and its variants in nuclear proteins from eukaryotes, each of the GRKTR and its variants was compared to protein sequences in the model organisms (landmark) database. Only soluble proteins predicted to localize in the nucleus according to current protein databases (the protein database from NCBI and The Human Protein Atlas [46]), or those that have been experimentally localized in the nucleus according to the published literature, were considered real hits. Only 100% sequence similarity and their corresponding E-value were recorded for all the BLAST hits.

As controls, a five amino acid random peptide and the NLS of the SV40 large T-antigen were entered in all the BLAST searches. The random peptide, GLVTV, was generated using the Sequence Manipulation Suite (SMS) [47] web tool available at https://www.bioinformatics.org/sms2 (accessed on 25 February 2022).

The prediction of the NLSs was performed using the NLS prediction program NLStradamus [48] (available at http://www.moseslab.csb.utoronto.ca/NLStradamus/ (accessed on 20 August 2022)) with a threshold score of 0.6. Multiple protein sequences were aligned using Clustal Omega [49] available at https://www.ebi.ac.uk/Tools/msa/clustalo/ (accessed on 20 August 2022).

### 2.2. Construction of Plasmids

The PHW2000-NP plasmid (strain A/PR8/1934/H1N1) was generously provided by Dr. Honglin Chen from The University of Hong Kong and Dr. Robert Webster from St Jude Children Research Hospital. A QuikChange^®^ Site-Directed Mutagenesis Kit (Stratagene, La Jolla, CA, USA; Catalog number: 200518) was used to generate mutations in the basic amino acids of NLS1 or NLS2 in the PHW2000-NP plasmid. The PHW2000-NP plasmid served as a template.

The plasmid encoding five green fluorescent protein (GFP) molecules in tandem, generated by cloning in frame four GFP cDNAs into the pEGFP-C3 vector, was a generous gift from Dr. Gergely L. Lukacs, McGill University [50,51]. To generate the 5GFP-NLS2 constructs, the synthetic DNA of NLS2 from the influenza A virus strain A/X-31 H3N2 containing adapters of the Bam HI restriction enzyme were annealed, and the annealed DNA fragments were ligated to the Bam HI site at the C-terminal coding sequence of 5GFP. A Q5^®^ Site-Directed Mutagenesis Kit (New England Biolabs, Ipswich, MA, USA) was used to generate variants of the NLSs in 5GFP-NLS2. The wild-type (WT) plasmid was used as the template to generate different variants.

The human NOP14 cDNA plasmid (RG208813) in the pCMV6-AC-GFP vector was purchased from OriGene Technologies, Inc. (Rockville, MD, USA). A Q5^®^ Site-Directed Mutagenesis Kit (New England Biolabs) was used to generate mutations in the basic amino acids in NLS2 and/or NOP14-Seq3 plasmids.

All primers used in this study are listed in Table 1. All constructs were confirmed by sequencing.

### 2.3. Cell Culture, Transfection, and Imaging of Transfected Cells

HeLa cells were maintained at 37 °C, and 5% CO_2_ in Dulbecco’s modified Eagle’s medium (DMEM) supplemented with 10% fetal bovine serum, 1% penicillin/streptomycin, 1% L-glutamine, and 1% sodium pyruvate. HeLa cells grown as monolayers on glass microscope coverslips were transfected with 5GFP or 5GFP-NLS2 plasmids using Lipofectamine 2000 (Invitrogen) according to the manufacturer’s instructions. Twenty-four hours after transfection, the cells were fixed with 3% paraformaldehyde in phosphate-buffered saline (PBS) for 15 min at room temperature. The cells were then washed with PBS three times and fixed with 3% paraformaldehyde. Finally, the coverslips were mounted onto microscope slides in the Prolong Gold antifade reagent containing 4,6-diamidino-2-phenylindole (DAPI).

For the transfection of the NP and its mutants, HeLa cells grown as monolayers on glass microscope coverslips were transfected with PHW2000-NP plasmid or PHW2000-NP carrying mutations in NLS1 or NLS2 using Lipofectamine 2000 according to the manufacturer’s instruction. PBS (control) or leptomycin B (Sigma-Aldrich, St. Louis, MO, USA) was added as indicated to a final concentration of 11 nm (5.5 ng/mL) 18 h post-transfection according to a published protocol [26]. Thirty hours after transfection, the cells were fixed with 3% paraformaldehyde in phosphate-buffered saline (PBS) for 15 min at room temperature. The cells were then washed with PBS three times, fixed with 3% paraformaldehyde, followed by 5 min of permeabilization with 0.2% Triton X-100 (Sigma-Aldrich, Catalogue number: T8787). For immunolabeling, coverslips were incubated in a blocking buffer (BF: PBS containing 2.5% bovine albumin serum (BSA) (Sigma-Aldrich, Catalogue number: T8787) and 10% goat serum (Sigma-Aldrich, Catalogue number: G9023) at room temperature for 1 h. After blocking, the cells were incubated with an anti-NP antibody (Acris, Catalogue number: AM01375PU) diluted at 1:1000 in BF for 1 h at room temperature. Next, the cells were washed gently three times at 10 min intervals with PBS and then incubated with the goat anti-mouse IgG (H + L) conjugated with Alexa Fluor 568 secondary antibody (Thermo Fisher Scientific, Waltham, MA, USA, Catalogue number: A-11004), diluted in BF, for 1 h at room temperature. Coverslips were then washed three times at 5 min intervals with PBS and mounted with the ProLong gold antifade reagent containing DAPI.

For the transfection of NOP14-GFP and its mutants, HeLa cells grown as monolayers on glass microscope coverslips were transfected with NOP14-GFP plasmid or NOP14-GFP carrying mutations in NLS2 and/or Seq3 using Lipofectamine 2000 according to the manufacturer’s instruction. After transfection, cells were fixed with 3% paraformaldehyde and prepared for immunofluorescence microscopy, as indicated above. An antibody against fibrillarin (Abcam, Catalogue number: Ab4566) was used to detect the nucleolus at 1:100 dilution in BF for 1 h at room temperature.

All samples were visualized using a Fluoview FV1000 confocal laser-scanning microscope (Olympus, Tokyo, Japan).

### 2.4. Quantification of Nuclear Import

The quantification of the nuclear import of the chimeric proteins was performed as described in [42]. Briefly, the mean intensity of a defined area in the nucleus was measured and divided by the mean intensity of the same size area in the cytoplasm from the same cell using ImageJ (National Institute of Health). The fluorescence of the nuclear envelope was not included in the quantification. After correction for background fluorescence, the results were expressed as the ratio of nuclear to cytoplasmic fluorescence (Fn/c) or the ratio of the nucleolar to nuclear fluorescence (Fnucleolus/n). Data were obtained from a total of 85–100 cells per experiment from three independent experiments. The Kruskal–Wallis’s test (GraphPad Software, Inc., La Jolla, CA, USA) was used to analyze the Fn/c results of cells transfected with NP and its mutant plasmids. All other results were analyzed by the one-way ANOVA test followed by Tukey’s test using the GraphPad Prism Software (GraphPad Software, Inc., La Jolla, CA, USA). All data are represented as the mean value ± standard error of the mean, and *p* < 0.05 was considered significant.

## 3. Results

### 3.1. NLS2 Contributes to the Nuclear Import of NP to the Same Extent as NLS1

**Figure 1:**
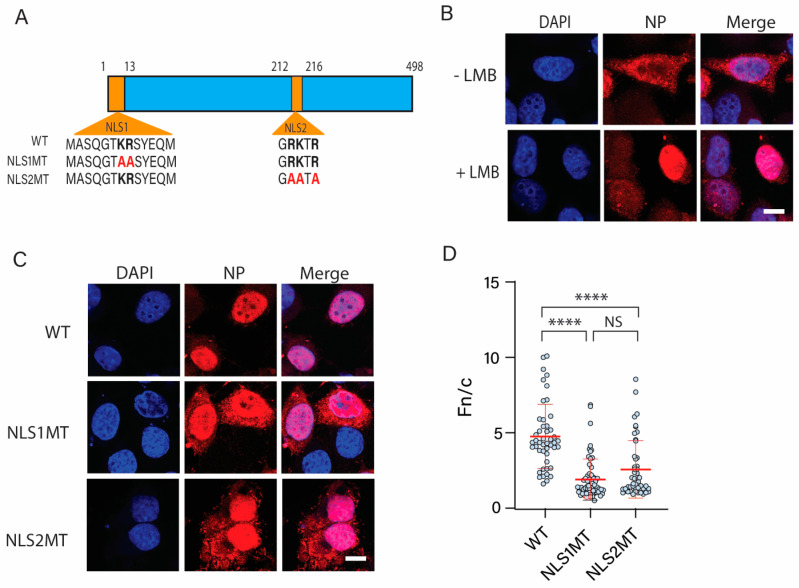
Subcellular localization of WT and mutant NPs. (**A**) Schematic representations of WT NP and its mutants (NLS1 MT and NLS2 MT). Basic residues of NLS1 and NLS2 are indicated in bold. Alanine substitutions of K or R are shown in red. (**B**) Confocal images of HeLa cells transfected with a plasmid expressing WT NP at 30 h post-transfection in the presence or absence of LMB. (**C**) Confocal images of HeLa cells transfected with plasmids expressing WT NP, NLS1, or NLS2 mutant NP at 30 h post-transfection in the presence of LMB. For B and C, LMB was added 12 h post-transfection to inhibit nuclear export of NP. Samples were prepared for indirect immunofluorescence microscopy using an antibody against NP (red), and nuclei were stained with DAPI. Scale bars for (**B**,**C**), 10 μm. (**D**) Quantification of the ratio of nuclear to cytoplasmic fluorescence (Fn/c) from the experimental conditions shown in (**C**). Shown is the means ± standard error of the means scored from 85–100 cells for each condition from three independent experiments. (NS, not significant; **** *p* < 0.0001, Kruskal–Wallis test).

To address the contribution of NLS2 to the nuclear import of NP, we performed mutagenesis and functional studies with the NP from a strain of influenza A virus (strain A/PR8/1934/H1N1) bearing the wild-type (WT) NLS2 sequence ^212^GRKTR^216^. An NP mutant construct was generated in which the basic residues of NLS2 were substituted for alanine (Figure 1A). Another NP construct with the basic residues of the NLS1 substituted for alanine was also generated (Figure 1A). Because the NP shuttles between the nucleus and the cytoplasm [52] and uses the cellular chromosome region maintenance 1 (CRM1) pathway for nuclear export [53], to study the NP nuclear import we used a protocol previously published [26] in which the NP nuclear export was blocked with leptomycin B (LMB), which inhibits the CRM1-dependent nuclear export [54]. The HeLa cells were transfected with the WT NP plasmid, LMB was added 12 h post-transfection at a final concentration of 11 nM, and the cells were fixed 18 h after the treatment. As a control, cells were mock-treated with PBS. As expected, LMB efficiently retained the NP in the nucleus of the cells transfected with the WT construct at 30 h post-transfection (Figure 1B). When the mutant NLS1 or NLS2 NP constructs were expressed in the HeLa cells in the presence of LMB, there was a strong NP fluorescence signal in the nucleus of the transfected cells (Figure 1C). Although, compared with the WT NP, there was some cytoplasmic localization for both mutant NPs, the NLS2 mutant showed similar nuclear localization as the NLS1 mutant NP (Figure 1C,D). The quantification of the nuclear to cytoplasmic fluorescence intensity ratio (Fn/c) of the NP indicates that the nuclear localization of the WT NP was significantly higher than that of the NLS1 and NLS2 NP mutants (Figure 1D). Thus, mutations of either NLS1 or NLS2 significantly reduce the nuclear accumulation of NP. These results emphasize that both NLS1 and NLS2 are indispensable and work in synergy in the nuclear import of NP when produced in the absence of other viral proteins or vRNA.

### 3.2. Five NLS2 Variants Are Present in NP of Different Influenza A Virus Strains, but Most Strains Contain the Wild-Type Sequence

As with the other NLSs, NLS2 may have natural variants in which the basic amino acids that bind to the importin-α major binding site are conservatively replaced. With five residues, ^212^GRKTR^216^ (designated P1, P2, P3, P4, and P5, according to their position at the major binding site of importin-α), and three basic amino acids, there are eight possible NLS2 variants (Table 2). We named the sequence ^212^GRKTR^216^, found in the NP from seasonal influenza A virus strains, the WT variant. The other seven variants were named according to the importin-α position where the conservative amino acid substitution occurs compared to the WT sequence (for example, R to K replacement at position 213 was named P2 variant; Table 2).

**Figure 2:**
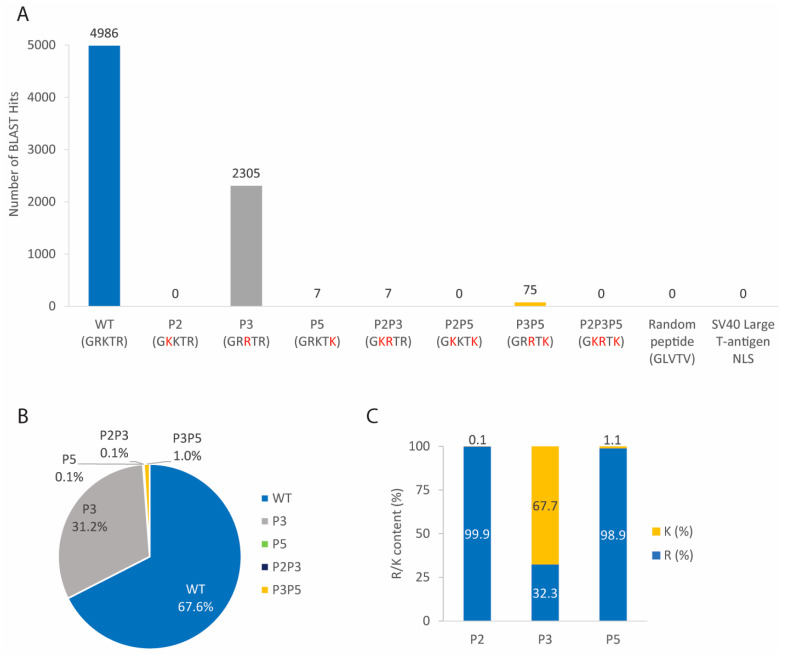
Bioinformatics of the sequence GRKTR and its variants for NP from different influenza A virus strains. (**A**) The sequence GRKTR and its variants were entered into the BLASTP sequence alignment algorithm, and the number of hits for each variant is reported for NP from influenza A viruses. As controls, a random peptide consisting of five amino acids (GLVTV) and the SV40 Large T-antigen NLS (PKKKRK) were also used in the BLASTP analysis. The amino acids in red indicate substitutions from the WT NLS2. (**B**) Percentage of NLS2 variants in NP from different strains of influenza A viruses. (**C**) Percentage of K and R at P2, P3, and P5 of NP from different influenza A virus strains.

We performed bioinformatics analyses using the Basic Local Alignment Search Tool for proteins (BLASTP) [45] to find whether NLS2 variants are naturally present in the NP from other influenza A virus strains and other influenza virus types. As negative controls, a random peptide consisting of five amino acids (GLVTV) generated by the Sequence Manipulation Suite (SMS) [47] and the NLS sequence of the SV40 Large T-antigen (PKKKRK) were also included in the analysis. The BLASTP results of 7380 NP sequences from different influenza A virus strains show that five of the eight NLS2 variants were present in the NP from different strains of the influenza A virus (Figure 2A). However, a similar analysis with the NP from influenza B viruses found only one hit for the WT sequence, present at residues 63–67 (^63^GRKTR^67^) of the NP from the influenza B virus strain B/New York/37/2016 (accession number: AOT97159.1). The BLASTP results of all NPs from influenza C and D viruses in the database revealed that proteins from these viruses did not contain either the WT NLS2 sequence or any of its variants. There were no hit sequences for the control peptides (the five amino acid random peptide and the NLS of the SV40 Large T-antigen) in the BLASTP results with all NPs from all influenza viruses.

Although five NLS2 variants were present in the NP of all influenza A virus strains in the database (Figure 2A), two variants were most abundant. The variant WT was the most abundant; 4986 NP sequences from influenza A viruses have this variant (Figure 2A), which corresponds to 67.6% of the total NP sequences in the database (Figure 2B). The second most abundant sequence was P3, which was present in 2305 NP sequences of influenza A viruses (Figure 2A); this is 31.2% of the total NP sequences in the database (Figure 2B). In contrast, the abundance of P5, P2P3, and P3P5 were at or below 1% of the total searched NP sequences (0.1%, 0.1%, and 1%, respectively; Figure 2B). The three variants that were absent were the single substituted P2, the double substituted P2P5 variant, and the triple substituted P2P3P5 (Figure 2A).

Further analyses regarding arginine and lysine content at each position show that R (present in WT) was more prominent than K at positions P2 and P5; 99.9% of NPs contain R at P2, and 98.9% of NPs have R at P5 (Figure 2C). However, K (present in WT) was more prominent than R at the P3 position (67.7% of K versus 32.3% of R; Figure 2C).

While 99% of influenza A NP sequences contain the NLS2 variants spanning residues 212–216, some NLS2 variants were found at different NP sequence locations. For example, the WT NLS2 was found at residues 218–222 of the NP from three swine strains (Table S1). The P3 variant was found to span either residues 218–222 of the NP for 50 swine and canine strains or residues 213–217 of the NP from the mallard strain and the American wigeon strain (Table S1).

In summary, although five of the eight NLS2 variants were found to occur naturally in the NP of different influenza A virus strains, only the WT and the P3 sequences were predominant and occurred in 98.8% of the total sequences. The location of the NLS2 variants at amino acids 212–216 of the NP was highly conserved among different influenza A strains, with only very few strains displaying WT and P3 at different locations.

### 3.3. The Five NLS2 Variants Present in NP of Influenza A Virus Mediate Nuclear Import of Chimeric Proteins

**Figure 3:**
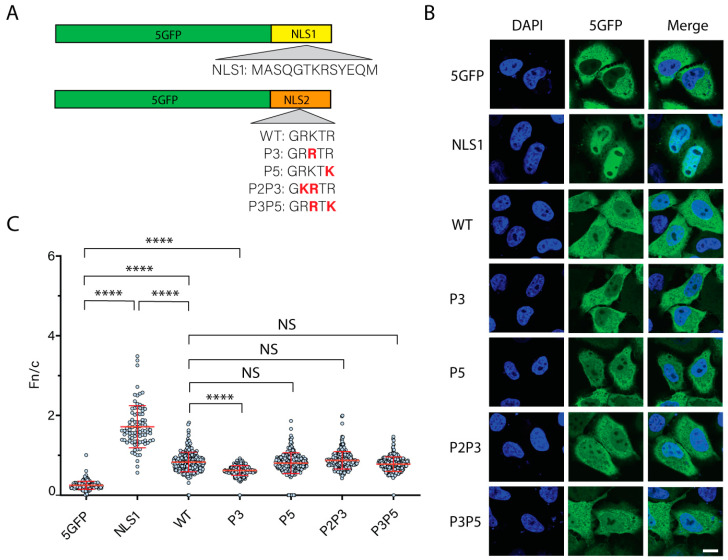
Functional analysis of NLS2 variants naturally found in NP of different influenza A virus strains. (**A**) Schematic representation of the chimeric proteins containing NLS1 and NLS2 variants fused to the C-terminus of 5GFP. The amino acids in red indicate substitutions from the WT NLS2 sequence. (**B**) Confocal images of HeLa cells transfected with plasmids expressing 5GFP, 5GFP-NLS1, or five NLS2 variants fused to 5GFP 24 h post-transfection. Nuclei were stained with DAPI. Scale bar, 10 μm. (**C**) Quantification of the ratio of nuclear to cytoplasmic fluorescence (Fn/c) from the experimental conditions shown in B. Shown is the means ± standard error of the means scored from 85–100 cells for each condition from three independent experiments. (NS, not significant; **** *p* < 0.0001, one-way ANOVA followed by Tukey’s tests).

It was previously shown that the WT sequence and the P3 variant were functional NLSs [28]. The chimeric protein localized to the nucleus when these sequences were fused to the C-terminus of five GFP molecules in tandem (5GFP) [28]. 5GFP was chosen because oligomers with four or fewer tandem copies of GFP freely diffused into the nucleus through the NPC, while 5GFP was excluded [51]. Although these two NLS2 variants mediated the nuclear import of the chimeric protein, the P3 variant yielded a lesser nuclear localization of the 5GFP chimeric protein than the WT sequence [28]. Thus, other variants may have a higher or lesser nuclear import efficiency than the WT sequence. To test this, we employed the same functional analyses used by Wu et al. [28] and transiently transfected the HeLa cells with plasmids encoding the five NLS2 variants found in the NP of influenza A viruses (Figure 2A) fused to the C-terminus of the 5GFP (Figure 3A). As controls, the HeLa cells were transfected either with a plasmid encoding the 5GFP or a plasmid encoding the NLS1 fused to the 5GFP (Figure 3A). The subcellular localization of the chimeric proteins was assessed 24 h post-transfection using confocal laser scanning microscopy. As expected, without an NLS, the 5GFP was in the cytoplasm of transfected cells (Figure 3B). In contrast to the 5GFP, the majority of the NLS1 chimeric protein localized predominantly in the nucleus of the transfected cells (Figure 3B).

Consistent with having a lower affinity for importin-α (the equilibrium binding constants of NLS1 and NLS2 are 4.9 μM and 72.4 μM, respectively [28], while this value is in the nanomolar range for cNLSs [55,56]), the nuclear accumulation of 5GFP-NLS2 WT was less than that of 5GFP-NLS1 (Figure 3C). However, all five NLS2 variants tested targeted the 5GFP to the nucleus, although at a lower extent compared to 5GFP-NLS1 (Figure 3B,C). The variants P5, P2P3, and P3P5 yielded a similar nuclear localization of the chimeric protein as the WT variant (Figure 3C). However, as previously reported [28], the nuclear accumulation of the chimeric protein containing the P3 variant was significantly lower than that of the WT sequence (Figure 3C).

The difference between Fn/c for 5GFP-NLS1 and 5GFP-NLS2 was significant (Figure 3C). This is in contrast to the results with the full-length NP containing NLS1/NLS2 mutations (Figure 1), for which the Fn/c for NP-NLS1MT and NP-NLS2MT was not significant (Figure 1D). Taken together, these results are consistent with the model that the NLS2 functions in synergy with NLS1, forming a bipartite NLS in the context of the full-length NP.

### 3.4. From All Viral Protein Sequences from DNA Viruses in the Database, NLS2 and Its Variants Are Present Only in a Few Viral Proteins

To find whether the sequence GRKTR and/or any of its variants are present in viral proteins from other viruses that replicate in the nucleus, each of the sequences listed in Table 2 was compared with the protein sequences from DNA virus families in the non-redundant database via the BLASTP. Only viral proteins reported in the literature to localize in the nucleus of infected cells experimentally were considered in this study. Moreover, because the putative NLS must be exposed to interact with importin-α for nuclear import, membrane proteins and proteins with resolved structures showing the NLS2 sequences not exposed on the protein structure were not considered.

From all DNA virus families analyzed, only seven proteins from the *Adenoviridae*, four proteins from the *Herpesviridae*, one from the *Circoviridae*, and one from the *Hepadnaviridae* families contained the sequence GRKTR or its variants (Table 3 and Table S3). All these proteins function in the nucleus of infected cells. For most of these proteins, the location of the GRKTR variant sequence seems to be conserved. For example, for the minor core protein pV, the P2P3 sequence is consistently present at residues 117–121 of several human adenovirus strains (Table 3).

As controls, the random peptide GLVTV and the NLS of SV40 large T-antigen were also compared to viral proteins from DNA virus families in the non-redundant database. We found that neither the random peptide nor the SV40 large T-antigen NLS was present in viral proteins from *Adenoviridae*, *Herpesviridae*, and *Hepadnaviridae* families. However, four hits of the random peptide sequence were found in the replicase protein of porcine circovirus 2. Further analysis of the BLAST expectation values (E-values), which measure the likeliness that sequence similarity happened by chance (with a smaller E-value indicating a higher quality of the match) [79,80], showed that the mean E-value for the four hits of the random peptide sequence found in the replicase protein of porcine circovirus 2 was 6.9 ± 0.0 and the E-value for the only hit of the P3 variant of the capybara associated cyclovirus 1 capsid protein was 2.2 (Table S2). The fact that the random peptide sequence resulted in no-hits for influenza A NP and proteins from DNA viruses, except for the *Circoviridae* family in which the E-value was significantly higher (*p* < 0.001, one-way ANOVA followed by Tukey’s tests) than the E-value for the studied sequences, indicates that the number of expected occurrences by chance is considerably small, and the alignment is truly meaningful.

**Figure 4:**
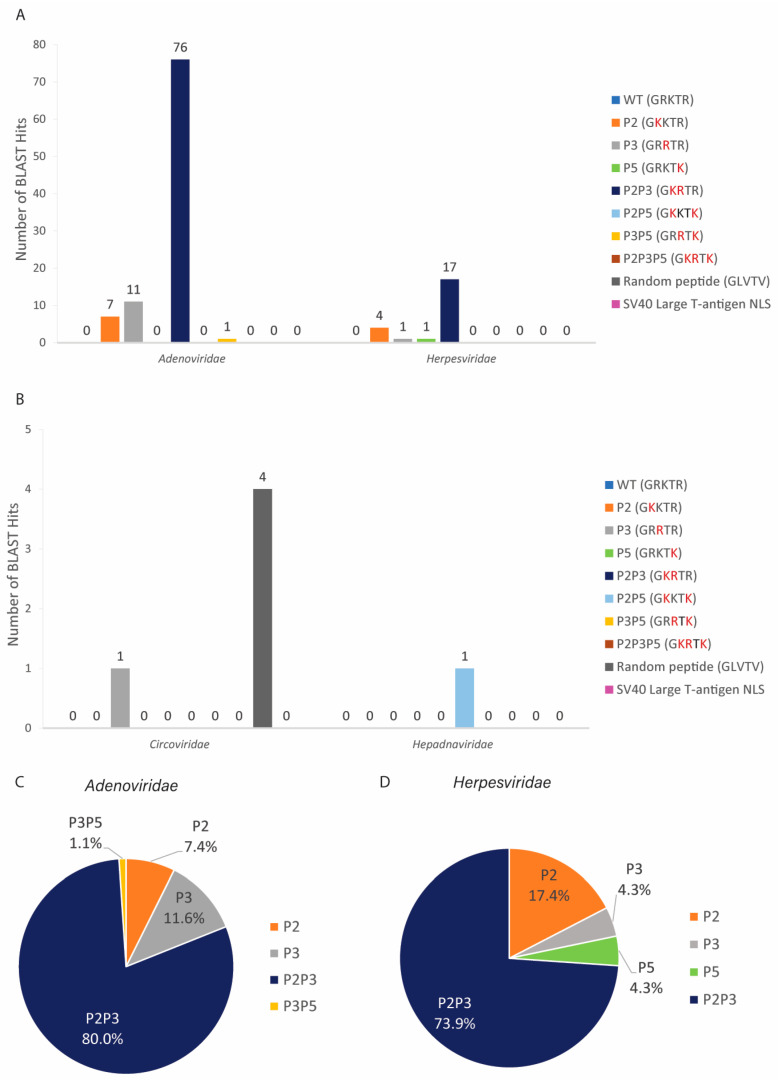
Bioinformatics of the sequence GRKTR and its variants present in proteins from DNA virus families that localize in the nucleus of infected cells. (**A**,**B**) The sequence GRKTR and its variants (listed in Table 2), the control peptide GLVTV, and the SV40 large T-antigen NLS were entered into the BLASTP algorithm. Only the numbers of hits for viral proteins that are known to function in the nucleus of infected cells are reported. The amino acids in red indicate substitutions from the WT NLS2. (**C**,**D**) Percentage of the sequence GRKTR and its variants present in the total viral proteins with these sequences from the *Adenoviridae* (**C**) and the *Herpesviridae* (**D**) families.

The *Adenoviridae* family contained the highest number of hits (95 BLAST hits from seven different proteins), followed by *Herpesviridae,* with 23 BLAST hits from four different proteins (Figure 4A**)**. The *Circoviridae* and *Hepadnaviridae* families each had one BLAST hit (Figure 4A). In contrast with influenza A NP, where the WT sequence was the most abundant (Figure 2B), the WT sequence was not found in any adenoviral or herpesviral proteins (Figure 4A). The P2P3 variant (which occurred only in 0.1% of influenza A NP sequences, Figure 2B) was the most abundant sequence in proteins from the *Adenoviridae and Herpesviridae* families (Figure 4C,D). In addition to the P2P3 sequence, three other sequences (P2, P3, and P3P5) were found in adenoviral proteins but at low abundance (Figure 4C). For herpesviral proteins, P2 was present in 17.4% of the total sequences with GRKTR variants (Figure 4D).

For influenza A NP, NLS2 and its variants synergize with NLS1 as a bipartite NLS [28]. Similarly, the sequence GRKTR and its variants may function together with an additional NLS in the viral proteins listed in Table 3. Thus, we used the NLS prediction program NLStradamus [48] to see if additional putative NLSs are present in the viral proteins we identified bearing the sequence GRKTR and its variants (Table 3). We found that three of the seven adenoviral proteins (the minor core protein pV, the precursor terminal protein pTP, and the viral RNA splicing factor L4-33 kDa) and three of the four herpes viral proteins (DNA polymerase processivity subunit, Tegument protein VP22, and Tegument protein UL32) have a second predictable NLS (Table 3).

Interestingly, the NLS2 variant sequences were included in the predicted NLS for some proteins. For example, the P3 sequence was found to overlap with the region containing the predicted NLS of the adenovirus L2 mu core protein pX (Table 3). For herpes virus proteins, the P3 and P2P3 variants shared the same region with the predicted NLS in tegument protein VP22 and assembly protein M80, respectively (Table 3). Moreover, the P3 sequence was also presented in the predicted NLS of the capybara-associated cyclovirus 1 capsid protein (Table 3).

In summary, a few viral proteins from four different DNA virus families (*Adenoviridae*, *Herpesviridae, Circoviridae*, and *Hepadnaviridae*) contain the putative NLS2 or its variant sequences and a second putative NLS. Although the WT variant was the most abundant sequence for influenza A NP (Figure 2B), this variant was not predominant for DNA viral proteins (Figure 4). The variants P2 and P2P5 did not exist in influenza A NP but were found in DNA viral proteins, and these NLS2 variants were able to nuclear import 5GFP (Figure S1). P2 was present in adenoviral and herpesviral proteins, and P2P5 was found in only one hepatitis B virus protein. Although the P2P3P5 was able to localize the GFP to the nucleus (Figure S1), it was not found in influenza A NP or any other viral proteins.

### 3.5. The Sequence GRKTR and Its Variants Are Present in a Very Low Proportion of Nuclear Proteins

**Figure 5:**
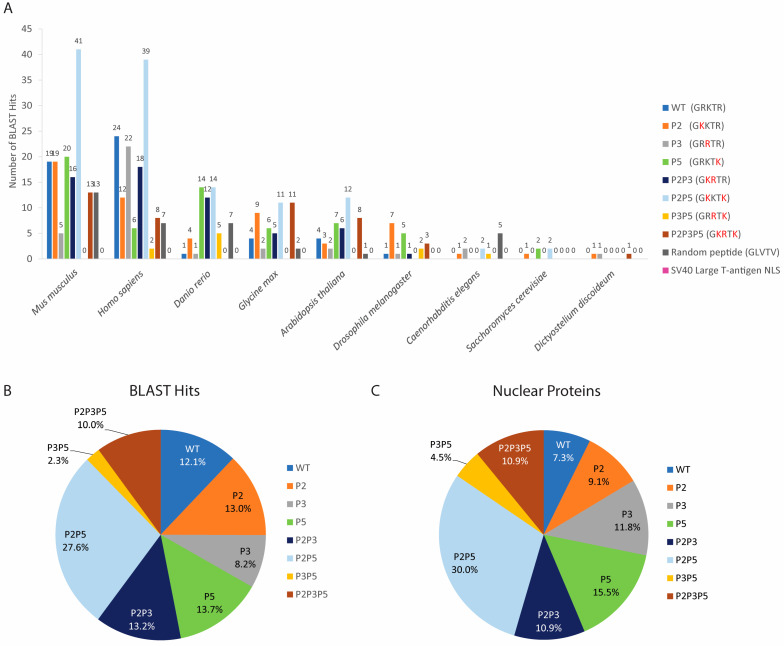
Bioinformatics of the sequence GRKTR and its variants for nuclear proteins. (**A**) The sequence GRKTR and its variants (listed in Table 2), the control peptide GLVTV, and the SV40 large T-antigen NLS were entered into the BLASTP algorithm. Only the number of hits for proteins that are known to localize in the nucleus of cells from humans and several model organisms is reported. The amino acids in red indicate substitutions from the WT NLS2. (**B**) Percentage of BLAST hits for nuclear proteins containing each of the studied sequences. (**C**) Percentage of nuclear proteins containing each of the studied sequences.

To determine whether cellular proteins known to function in the nucleus contain the sequence GRKTR and/or any of its variants, each of the sequences listed in Table 2 was aligned against soluble proteins that have been experimentally shown to localize in the nucleus of cells from humans and several model organisms in the landmark database via the BLASTP. The random control peptide (GLVTV) and the SV40 large T-antigen NLS were also aligned against these nuclear proteins. The results show that all eight GRKTR variant sequences were present in nuclear proteins, with the P2P5 sequence being the most abundant (Figure 5). While none of the searched nuclear proteins contained the SV40 large T-antigen NLS, 35 BLAST hits for the control peptide were found in 10 nuclear proteins (Figure 5A). However, all BLAST E-values for the GRKTR sequence and its variants were significantly lower than the E-value for the control peptide (Table S6), indicating that the database match was less likely a result of random chance.

A total of 439 BLAST hits for NLS2 and its variants were found in 107 different nuclear proteins from different organisms (Table 4,  Tables S4 and S5). Mouse proteins (*Mus musculus*) had the highest number of BLAST hits (133 hits from 32 proteins), followed by human (131 hits from 28 proteins), zebrafish (*Danio rerio*; 51 hits from 17 proteins), soybean (*Glycine max*; 48 hits from 21 proteins), thale cress (*Arabidopsis thaliana*; 42 hits from 21 proteins), and fruit fly (*Drosophila melanogaster*; 20 hits from 8 proteins) (Figure 5A). A few BLAST hits were found for the nematode *Caenorhabditis elegans* (six hits from six proteins), the budding yeast *Saccharomyces cerevisiae* (five hits from five proteins), and the cellular slime mold *Dictyostelium discoideum* (three hits from three proteins) (Figure 5A).

Although there was a total of 439 BLAST hits (Figure 5A), the proportion of nuclear proteins containing the GRKTR sequence or its variants in each organism was very low. For example, in mice, which contained the highest number of proteins with the studied sequence, from the 3567 mouse nuclear proteins [96], there were only 32 proteins with the GRKTR sequence or its variants, which accounts for only 0.90% of mouse nuclear proteins. Similarly, out of 6758 human nuclear proteins [46], only 28 proteins contained the studied sequences (Table 4), corresponding to only 0.41% of the total human nuclear proteins. For Drosophila (about 4000 nuclear proteins identified in embryo nuclei by mass spectrometry [97]) and *S. cerevisiae* (total nuclear proteins 1515 proteins [98]), this number was 0.20% and 0.33%, respectively. The latter highly contrasts with the 56.7% of *S. cerevisiae* nuclear proteins containing cNLS sequences [3]. Thus, the GRKTR sequence is not as common as the cNLS in nuclear proteins.

Notably, several nuclear proteins with putative NLS2 or its variants have predictable NLSs (Table 4, Tables S4 and S5). Thus, depending on the structure of these proteins, NLS2 might function in synergy with the predicted NLS. In addition, 8 of the 28 human nuclear proteins do not have a predictable NLS. Moreover, the sequence GRKTR or its variants overlap with some of the predicted NLSs (Table 4, Tables S4 and S5). For example, the predicted NLSs of ten human nuclear proteins have overlapping regions with the GRKTR variants (Table 4 and Table S4).

Nuclear proteins containing the studied sequences found in humans and in one or more other organisms are listed in Table 4. Interestingly, three of these proteins localize in the nucleolus: NOP14, nucleolin, and DnaJ homolog subfamily C member 21 (DNAJC21). NOP14 from humans and mice, which plays an essential role in processing pre-18S rRNA and in assembling the small ribosomal subunit [81], contains the WT sequence (Table 4). Nucleolin from humans, mice, and soybeans, which is involved in pre-rRNA transcription and ribosome assembly [99], contains the P2P5 sequence (Table 4). Both mouse and human DNAJC21, which plays a role in the maturation of the ribosome 60S subunit [90], also contain the P2P5 sequence (Table 4). Other proteins listed in Table 4 are spliceosomal and replication factors, DNA-binding proteins, and various transcription factors.

### 3.6. Identification of a Novel NLS in Nucleolar Protein 14

**Figure 6:**
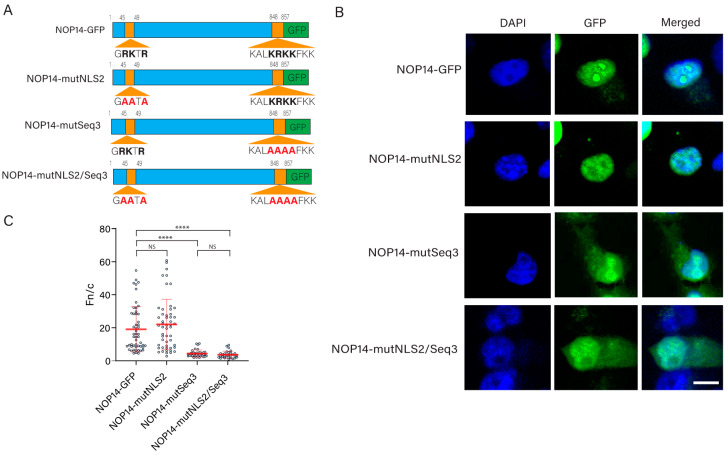
Functional analysis of NLS2 and a predicted NLS of NOP14. (**A**) Schematic representation of the chimeric proteins NOP14-mutNLS2, NOP14-mutSeq3, and NOP14-mutNLS2/Seq3 fused to GFP. Basic residues of NLS2 and Seq3 are indicated in bold. The amino acids in red indicate substitutions from the WT NOP14 sequence. (**B**) Confocal images of HeLa cells transfected with plasmids expressing the proteins shown in A 24 h post-transfection. Nuclei were stained with DAPI. Scale bar, 10 μm. (**C**) Quantification of the ratio of nuclear to cytoplasmic fluorescence (Fn/c) from the experimental conditions shown in B. Shown is the means ± standard error of the means scored from 85–100 cells for each condition from three independent experiments. (NS, not significant; **** *p* < 0.0001, one-way ANOVA followed by Tukey’s tests).

To validate one of the bioinformatics hits, we chose to study NOP14 because its NLSs have not yet been characterized, and the NLS2 of the influenza A virus NP is involved in the nucleolar localization of NP [44]; WT NP, but not a mutant NP with alanine substitutions in NLS2, localizes to the nucleolus at 12 h post-transfection [44]. To determine whether the sequence ^45^GRKTR^49^ is an NLS of NOP14, we designed a construct that contains alanine substitution of the basic amino acids of the GRKTR motif in a plasmid encoding NOP14-GFP (NOP14-mutNLS2) (Figure 6A) and used it to transfect the HeLa cells. As controls, the HeLa cells were transfected with a construct encoding NOP14-GFP (Figure 6A). The subcellular localization of the chimeric proteins was assessed 24 h post-transfection using confocal laser scanning microscopy. We found that NOP14-mutNLS2 yielded a similar nuclear localization of the chimeric protein as NOP14-GFP (Figure 6B,C), indicating that the replacement of basic residues by alanine in the sequence ^45^GRKTR^49^ did not affect the nuclear import of NOP14.

**Figure 7:**
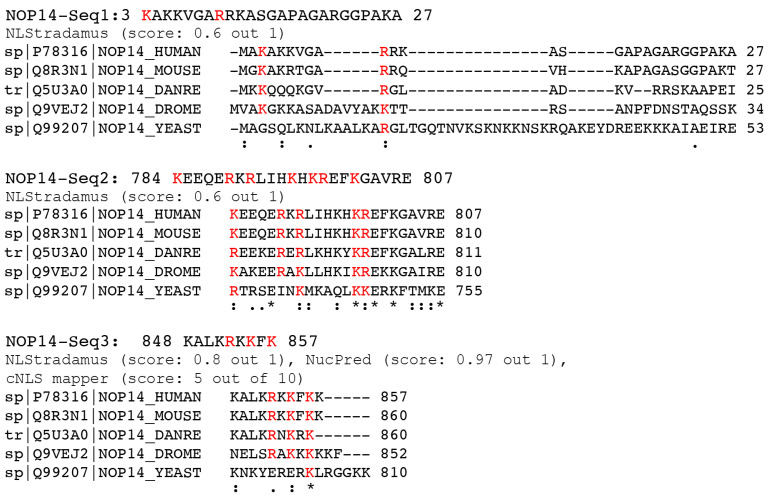
Protein sequence alignments of the putative NLSs of NOP14 from five different organisms. The conserved basic residues are in red. The sequences are from humans, mouse, zebrafish (*Danre*), fruit fly (*Drome*), and yeast. An asterisk (*) indicates positions that have single, fully conserved residues in all five sequences; a colon (:) indicates conserved amino acids of strongly similar properties; and a period (.) indicates conservation between amino acids of weakly similar properties.

Because the mutation of NLS2 did not abolish the nuclear import of NOP14, this protein may possess other functional NLSs. According to our NLS prediction analysis (Table 4), NOP14 has three putative NLSs which were predicted using NLStradamus [48]. To further consider these putative NLSs, we evaluated their conservation using Clustal Omega [49] to align the predicted NLSs from five different organisms (human, mouse, zebrafish, fruit fly, and yeast) (Figure 7). Although the basic residues of both NOP14-Seq2 and NOP14-Seq3 are highly conserved, NOP14-Seq1 contained multiple gaps in sequence alignments (Figure 7) and was, therefore, eliminated for further functional studies.

We also examined the predicted NLSs of NOP14 with two other NLS prediction software tools, the cNLS mapper [100] and NucPred [101], and found that Seq1 and Seq2 were only predicted by NLStradamus with a medium score (0.6 out 1) (Figure 7). However, Seq3 was predicted by all three different NLS prediction software and had very high scores for NLStradamus (0.8 out of 1) and NucPred (0.97 out of 1) (Figure 7). Thus, from the three sequences, Seq3 is very likely to be a functional NLS of NOP14. Therefore, we chose to study Seq3.

To determine whether Seq3 is a functional NLS of NOP14, we designed a construct encoding NOP14-GFP that contains alanine substitutions of the four central basic amino acids of Seq3 (Figure 6A). In addition, a construct in which both ^45^GRKTR^49^ and Seq3 were mutated was also generated (Figure 6A). When these plasmids were transfected into HeLa cells, the nuclear accumulation of the chimeric proteins containing the NOP14-mutSeq3 and the double mutant NOP14-mutNLS2/Seq3 were significantly lower than that of NOP14-GFP (Figure 6B,C). These results demonstrated that NLS2 is not an NLS of NOP14, but Seq3 (^848^KALKRKKFK^857^) is a functional NLS of NOP14.

### 3.7. NLS2 Plays a Role in the Nucleolar Localization of Nucleolar Protein 14

**Figure 8:**
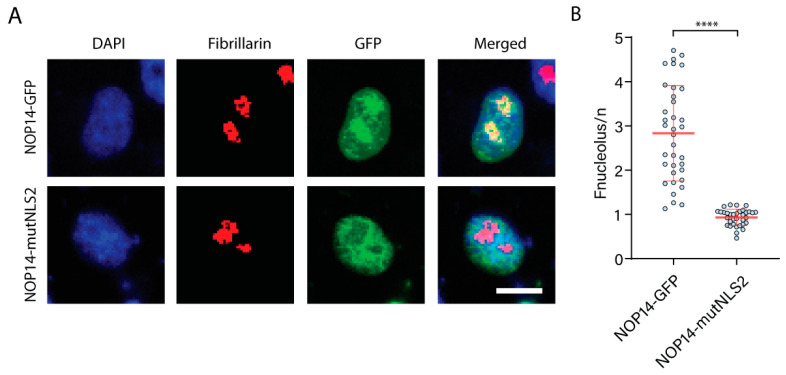
NLS2 plays a role in the nucleolar localization of NOP14. (**A**) Confocal images of HeLa cells transfected with plasmids expressing NOP14-GFP or NOP14-mutNLS2 24 h post-transfection. Samples were prepared for indirect immunofluorescence microscopy using an antibody against fibrillarin (red), and nuclei were stained with DAPI. Scale bar, 10 μm. (**B**) Quantification of the ratio of nucleolar to nuclear fluorescence (Fnucleolus/n) from the experimental conditions shown in A. Shown are the means ± standard error of the means scored from 50–80 cells for each condition from three independent experiments. (**** *p* < 0.0001, one-way ANOVA followed by Tukey’s tests).

We noticed that the mutations in NLS2 affected the nucleolar localization of NOP14 (Figure 6A). This observation is particularly interesting because NLS2 plays a role in the nucleolar localization of both progeny NP during infection and exogenous NP produced in tissue culture cells [43,44]. To prove that the nucleolar localization of the NP was dependent on NLS2, we examined the colocalization of NOP14-GFP and NOP14-mutNLS2 with a nucleolar marker (fibrillarin). The HeLa cells were transfected with constructs encoding NOP14-GFP or NOP14-mutNLS2, and the subcellular localization of the chimeric proteins and fibrillarin were assessed 24 h post-transfection using confocal microscopy (Figure 8A). We found that, indeed, the replacement of basic residues by alanine in the sequence ^45^GRKTR^49^ significantly reduced the nucleolar localization of NOP14 (Figure 8B). There was still detectable NOP14 colocalized with fibrillarin in cells expressing NOP14-mutNLS2 (Figure 8A), indicating that NLS2 is necessary but not sufficient to mediate the nucleolar localization of this protein.

## 4. Discussion

The Influenza A virus NP has a newly defined monopartite NLS at residues 212–216 (^212^GRKTR^216^), termed NLS2 [28]. In this study, we first demonstrated that NLS2 is critical for the nuclear import of NP. Next, we performed bioinformatics of NLS2 variants with conservative replacements of the three basic amino acids and studied their function. We found that only five of the eight NLS2 variants are present in the NP from different influenza A virus strains, although all eight NLS2 variants were nuclear transport-efficient in our functional studies. We also demonstrated that the GRKTR sequence and its variants are present in only a few viral proteins that function in the nucleus of infected cells and in a very low proportion of nuclear proteins from different organisms.

NLS2 variants were present in the NP from all influenza A viruses and only in one strain of influenza B virus, but not in the NP from influenza C and D viruses. The GRKTR sequence or its variants were also not present in other proteins from influenza or other viruses of the *Orthomyxovirus* family. Thus, within the *Orthomyxoviruses,* NLS2 is an exclusive sequence of NP from the influenza A virus. Five of the variants transported GFP into the nucleus with efficiency similar to that of WT NLS2 (Figure 3 and Figure S1). The exceptions were the P3 and P2P3P5 variants that targeted less 5GFP to the nucleus than the WT sequence (Figure 3 and Figure S1). However, only two variants (WT and P3) were predominantly found in 98.8% of NP sequences in the database; three other variants (P3, P2P3, and P3P5) accounted for the remaining 1.2% of the total reported sequences (Figure 2B). Even though there was a very small variation of the NLS2 location in the amino acid sequence of the NP from different influenza A virus strains, this sequence was highly conserved and spanned residues 212–216 of the NP. This agrees with the characterization of NP as a highly conserved viral protein [102,103] and contrasts with the influenza envelope proteins, hemagglutinin, M2, and neuraminidase, which mutate easily [104,105]. Thus, the influenza A virus would be less prone to develop resistance to NP inhibitors. The high level of conservation of the NLS2 location in NP and the presence of either the WT NLS2 or its variant P3 in 99% of the NP sequences in the database suggests that NP inhibitors targeting NLS2 could be potent antivirals.

Structural studies by X-ray crystallography of several peptides in complex with importin-α have precisely mapped the interaction of several NLSs with importin-α (reviewed in [106]). The five contact points for the NLS peptides with the major NLS-binding site of importin-α have been termed P1–P5 [9,19]. K at the P2 position is highly conserved in all well-characterized cNLS sequences (Table 5). The substitution of K to non-basic amino acids at P2 in the SV40 T-antigen cNLS completely abolishes the nuclear import of this protein [107,108]. Thus, P2 has been defined as the most critical position in the cNLS [106]. Although K is commonly at P2 and is conserved among cNLSs (Table 5), the two predominant NLS2 variants found in influenza A NP have R at P2 (Figure 2). From the studies with the cNLS, we may expect that NLS2 with K at P2 yields more nuclear accumulation of 5GFP than the WT sequence. However, this was not the case, as NLS2 variants with K at P2 yielded a similar nuclear import of 5GFP to that of the WT sequence (Figure 3 and Figure S1). Thus, as previously suggested, residues surrounding P2 may also be critical for the function of NLS2 [28]. Remarkably, NLS2 is not the only known NLS with R at P2; other examples of such NLS have been found in the capsid-associated protein of the circovirus beak and feather disease virus (BFDV) (Table 5) [109] and the p10 protein of the Borna disease virus [110].

While K at position P2 is highly conserved among cNLSs, P3 is more flexible and can accommodate a large variety of amino acids, including K, R, methionine (M), glutamate (E), or isoleucine (I) (Table 5). For influenza A NP, however, K is preferred over R at P3 (Figure 2C), and the P3 variant with R at P3 was the least efficient NLS variant in our functional assay (Figure 3C). Thus, in contrast to the cNLS, the presence of K at P3 is critical for the function of NLS2.

The majority of cNLSs contain K at P5 (Table 5). However, 98.9% of searched sequences for influenza A NP contained R at P5 (Figure 2C). Another example of an NLS with R at P5 is the NLS of the capsid-associate protein of BFDV (Table 5). Out of 7380 NP sequences from different influenza A virus strains, only seven sequences contained K at P5 (variant P5), and 75 other sequences had both K at P5 and a substitution at P3 (variant P3P5) (Figure 2A). It is expected that K at P5 might promote more nuclear import of the 5GFP because most of the well-characterized cNLS containing K are at this position. However, the nuclear import efficiency when the R at the P5 of NLS2 was changed to K (P5 and P3P5 variants) was similar to that of WT (Figure 3C). Thus, as suggested for the P2 site of NLS2 [28], residues neighboring P5 may also be important to maintain a strong interaction between NLS2 and importin-α.

Although we searched the database for proteins from all DNA viruses, we found that only a few proteins contain the GRKTR sequence or its variants (Figure 4). Moreover, the total number of reported hits was relatively low compared to those of influenza A NP (7380 BLAST hits): *Adenoviridae*, 95 BLAST hits; *Herpesviridae*, 23 BLAST hits; *Circoviridae*, one BLAST hit; and *Hepadnaviridae*, one BLAST hit. NLS2 variants were found only in seven adenoviral and four herpesviral proteins (Table 3). Three adenoviral proteins do not have predictable NLS (Table 3); thus, it is possible that the putative NLS2 functions to transport these proteins into the nucleus. However, whether these putative NLS2 sequences work as a signal to import these viral proteins into the nucleus of infected cells remains to be determined. Some of these sequences may not be exposed on the protein surface and/or would not interact with importin-α. For example, the VP1 capsid protein of parvovirus contains the P2P3 variant in its sequence (accession number: ABB01354), but this region has been shown to not function as an NLS for VP1 [111].

The GRKTR sequence and its seven variants were present in several nuclear proteins from different organisms (Figure 5, Table 4, Tables S4 and S5). However, the proportion of nuclear proteins containing the GRKTR sequence and its variant in each organism was very low (for example, only 0.41% of human nuclear proteins contained the studied sequence). Thus, the GRKTR sequence is not as commonly found in the nuclear proteins of humans or other organisms as the cNLS [3]. Nevertheless, the studied sequences were conserved across different species in some of these proteins. For example, the P2 variant was found in the replication factor C subunit 3 of seven different organisms (Table 4), and P2P5 was present in the nucleolin from three different organisms (Table 4). Only 66 nuclear proteins with putative NLS2 contained predictable NLSs (Table 4, Tables S4 and S5). The remaining 41 (out of 107) proteins do not have predictable NLSs (Table 4, Tables S4 and S5), and many of these are human proteins that are of particular interest due to their role in the response to disease and infection. For example, the transcription factor Sp1 is involved in the transcriptional control of the human FE65 gene, which encodes an important adaptor protein that binds to the Alzheimer’s disease amyloid precursor protein [112]. For the nuclear proteins that have both predicted NLSs and putative NLS2, it is important to consider that the GRKTR and its variant sequences may function in synchrony with the predicted NLS as the NLS1 and NLS2 of influenza A NP [28].

Because previous studies show a role of NLS2 in the nucleolar localization of NP [43,44], we chose NOP14, one of the nuclear protein hits containing the WT NLS2 sequence (Table 4), to study the functionality of NLS2 in this protein. Although the results indicated that the WT NLS2 found at the N-terminus of NOP14 is not a functional NLS, we identified a novel NLS located at the C-terminus of this protein (Figure 6). NLS2 at the N-terminus of NOP14 might work in synergy with the NLS at the C-terminus of NOP14. However, the experiment with the double mutant (Figure 6B,C) did not support this possibility.

That NLS2 is not a functional NLS of NOP14 strengthens our conclusion that NLS2 is a highly conserved NLS of influenza A NP. Interestingly, we found that similar to the nucleolar localization role of NLS2 for influenza NP [43,44], NLS2 also plays a role in the nucleolar localization of NOP14 (Figure 8). However, this protein may also have other NoLSs because nucleolar localization of NOP14-mutNLS2 decreased but was not abolished (Figure 8). Alternatively, NLS2 might be part of a larger NoLS of NOP14. Nevertheless, it is compelling that NLS2 has a nucleolar localization role in both influenza NP and human NOP14. Thus NLS2 might also have nucleolar localization roles in other proteins identified in our bioinformatics analyses. In agreement with this suggestion, the sequence ^380^RRRRRR**RRTR**^389^, which contains the P3 motif, was recently identified as one of the NoLS of the bovine adenovirus-3 protein V [58].

In conclusion, we used bioinformatics to demonstrate the natural existence of several NLS2 variants of influenza A virus NP. Although all eight variants are functional signals that mediate the nuclear import of 5GFP, only the variants WT and P3 were present in 98.8% of the total NP searched sequences (68% for WT and 30% for P3). The GRKTR sequence and its variants were also found in a few proteins from DNA viruses and in a very low proportion of nuclear proteins from various model organisms. Further studies will focus on determining whether the GRKTR sequence and its variants function as NLSs in the nuclear import of these proteins. Our study highlights that coupling bioinformatics and functional studies is a promising approach for identifying new NLSs in viral and cellular proteins.

## Figures and Tables

**Table 1:** Primers used in this study.

Construct Name	Primer Sequence (F Indicates Forward; R Indicates Reverse)
NLS1 MT	F′5′-GATCCAATGGCGTCTCAAGGCACCAAACGATCATATGAACAATGCCG-3′ R′5′-GATCCGGCATTTGTTCATACGATCGTTTGGTGCCTTGAGACGCCATTG-3′
NLS2 MT	F′5′-GAGGGGTGAAAATGGAGCAAAGACAGCGCCGAATC-3′ R′5′-GATCCGGCGCTGTCTTTGCTCCATTTTCACCCCTC-3′
NOP14-mutNLS2	F′5′-ACGGCCCACGACGTGGGACTGCCC-3′ R′3′-CGCCGCGCCCAGGATCTGGAACTTCTG-5′
NOP14-mutSeq3 and NOP14-mutNLS2/Seq3	F′5′-TCTGGCGGCGGCAGCGTTCAAAAAAACGCG-3′ R′3′-GCCTTCCATTCGCCTTCC-5′
P2	F′5′-TGAAAATGGAAAAAAGACAAGGCCG-3′ R′3′-CCCCTCCAGAAATTTCGG-5′
P3	F′5′-AAATGGACGAAGGACAAGGCCGG-3′ R′3′-TCACCCCTCCAGAAATTTCG-5′
P5	F′5′-ACGAAAGACAAAGCCGGATCCAC-3′ R′3′-CCATTTTCACCCCTCCAGAAATTTC-5′
P2P3	F′5′-TGAAAATGGAAAAAGGACAAGGCCG-3′ R′3′-CCCCTCCAGAAATTTCGG-5′
P2P5	F′5′-TGAAAATGGAAAAAGGACAAGGCCG-3′ R′3′-CCCCTCCAGAAATTTCGG-5′
P3P5	F′5′-ACGAAGGACAAAGCCGGATCCAC-3′ R′3′-CCATTTTCACCCCTCCAGAAATTTC-5′
P2P3P5	F′5′-AAATGGAAAAAGGACAAAGCCGG-3′ R′3′-TCACCCCTCCAGAAATTTC-5′

**Table 2:** Name of NLS2 variants.

NLS2 Variant	Position at the Importin-α Major Binding Site ^1^ P_1_ P_2_ P_3_ P_4_ P_5_
WT	^212^G R K T R^216^
P2: R→ K at 213	^212^G **K** K T R^216^
P3: K→ R at 214	^212^G R **R** T R^216^
P5: R→ K at 216	^212^G R K T **K**^216^
P2P3: R→ K at 213 and K→ R at 214	^212^G **K R** T R^216^
P2P5: R→ K at 213 and R→ K at 216	^212^G **K** K T **K**^216^
P3P5: K→ R at 214 and R→ K at 216	^212^G R **R** T **K**^216^
P2P3P5: R→ K at 213 and K→ R at 214 and R→K at 216	^212^G **K R** T **K**^216^

^1^ Substituted amino acids are in red.

**Table 3:** Viral proteins from DNA virus families containing variants of the sequence GRKTR and their predicted NLSs.

Protein ^1^	Protein Function ^2^ [References]	Virus/Host	Putative NLS2 Variant	Predicted NLS ^3^
***Adenoviridae* family**				
Minor core protein pV	Participates in capsid assembly in the nucleus [57,58]	Harbour porpoise adenovirus 1	P2: ^130^GKKTR^134^	^21^RKRKTPKREPKTEIKIERVKTEDVKPFKKGKRRKH^55^
		Human mastadenovirus B and several human adenoviruses (16, 3 + 7, 68, 66, 7d2)	P2P3: ^117^GKRTR^121^	^297^YKPPKRQYRKRKTRRVRQGRR^317^
Precursor terminal protein pTP	Participates in viral replication [59,60]	Titi monkey adenovirus ECC-2011	P3: ^350^GRRTR^354^	^339^GARPGLRRRPTAGRR^353^
		Squirrel monkey adenovirus	P3: ^351^GRRTR^355^	^389^RLPIRRRRRRAPP^401^
Viral RNA splicing factor L4-33 kDa	Required for genome packaging and capsid assembly in the nucleus [61,62]	Deer mastadenovirus B and murine adenovirus 3	P2: ^74^GKKTR^78 108^GKKTR^112^	^126^RGRRR^130^
Late L2 mu core protein pX	Condenses the viral pro-chromatin for encapsidation (reviewed in [63,64])	Murine adenovirus 2 and canine adenovirus 1	P3: ^26^GRRTR^30^ ^31^GRRTR^35^	^15^RSRRLRRRLGGGGCSSGRRTRRRSYRRRRGLR^46^
Encapsidation protein L1-52/55 kDa	Involved in genome packaging in the nucleus [65,66]	Duck adenovirus 4	P3: ^23^GRRTR^27^	No NLS predicted
Hexon-associated structural protein pVIII precursor	Capsid assembly in the nucleus by connecting the major structural units with each other and with the viral core (reviewed in [67,68,69])	Bovine adenovirus 1	P3P5: ^111^GRRTK^115^	No NLS predicted
Viral transcription factor L4-22 kDa	Required for genome packaging and capsid assembly in the nucleus [61,62]	Murine adenovirus 3	P2: ^84^GKKTR^88^	No NLS predicted
***Herpesviridae* family**				
DNA polymerase processivity subunit	Involved in viral DNA replication [70]	Wood mouse herpesvirus, murid gammaherpesvirus 4, and 68	P2: ^210^GKKTR^214^	^373^KRPPPKKEKEPTPKRPK^389^
Tegument protein UL32	Associates with nuclear capsids prior to DNA encapsidation and preserves the integrity of capsids through secondary envelopment [71,72]	Cynomolgus macaque cytomegalovirus strain Ottawa	P5: ^312^GRKTK^316^	^546^PKAKRRLILKPKTKKNVPKPKP^567^
Tegument protein VP22	Regulates the activity of the viral endonuclease vhs [73]	Pteropus lylei-associated alpha herpesvirus	P3: ^148^GRRTR^152^	^89^RRGRGAARPAAARAPTARRAPASGGAASARGTRGAAAS^126^ ^144^ASASGRRTRRP^154^
Assembly protein M80	Coordinates capsid assembly in the nucleus [74,75]	Murine betaherpesvirus 1	P2P3: ^504^GKRTR^508^ ^505^GKRTR^509^ ^506^GKRTR^510^ ^507^GKRTR^511^ ^508^GKRTR^512^	^507^GGKRTRQRGSADSGRKRRRRG^527^
***Circoviridae* family**				
Capsid protein	Binds and transports the viral genome through the NPC [76,77]	Capybara-associated cyclovirus 1	P3: ^9^GRRTR^13^	^5^RRFKGRRTRLPWRRSRFVRRRRGRFSRRTRRNYRR^39^
***Hepadnaviridae* family**				
X protein	Regulates transcription through direct interaction with different transcription factors [78]	Human hepatitis B virus	P2P5: ^124^GKKTK^128^	No NLS predicted

^1^ Accession numbers of the proteins are listed in Table S3. ^2^ All listed proteins are known to function in the nucleus of infected cells. ^3^ NLSs were predicted using NLStradamus [48]. Overlapping regions of the predicted NLS containing GRKTR or its variants are highlighted in red.

**Table 4:** Human nuclear proteins containing GRKTR variants found in one or more other organisms and their predicted NLSs.

Protein ^1^ [Localization References]	Organism	Putative NLS2 Variant	Predicted NLS ^2^
Nucleolar protein 14 (NOP14) [81]	*H. sapiens* *M. musculus*	WT: ^45^GRKTR^49^	^3^KAKKVGARRKASGAPAGARGGPAKA^27^ ^784^KEEQERKRLIHKHKREFKGAVRE^807^ ^848^KALKRKKFKK^857^
Nuclear mitotic apparatus protein 1 isoform X1 [82]	*H. sapiens* *M. musculus*	WT: ^1819^GRKTR^1823^ ^1805^GRKTR^1809^ ^1801^GRKTR^1805^ ^1797^GRKTR^1801^ ^1787^GRKTR^1791^ ^1783^GRKTR^1787^ ^1748^GRKTR^1752^ ^1734^GRKTR^1738^ ^1705^GRKTR^1709^	^2083^RRGASKKALSKASP^2096^ ^2127^AKGKAKH^2133^
Spliceosomal factor RED [83]	*H. sapiens* *M. musculus* *D. rerio* *A. thaliana*	WT: ^532^GRKTR^536^ ^513^GRKTR^517^ ^504^GRKTR^508^	^73^RRRKKKS^79^, ^541^KRK^543^ ^294^RNKKLKKKDKGKLEEKKP^311^ ^334^RDKERERYRERERDRERDRDRDRERERERDRERERERDREREEEKKRH^381^
Serum response factor [84]	*H. sapiens* *M. musculus* *D. rerio*	P2: ^137^GKKTR^141^ ^133^GKKTR^137^ ^116^GKKTR^120^	^135^KPGKKTRGRVKIK^146^
Replication factor C subunit 3 [85]	*H. sapiens* *M. musculus* *D. rerio* *D. melanogaster* *C. elegans* *S. cerevisiae* *D. discoideum*	P2: ^49^GKKTR^53^ ^48^GKKTR^52^ ^47^GKKTR^51^ ^46^GKKTR^50^	No NLS predicted
Transcription factor Sp1 [86]	*H. sapiens* *M. musculus*	P3: ^596^GRRTR^600^ ^594^GRRTR^598^ ^589^ GRRTR^593^ ^587^ GRRTR^591^ ^548^ GRRTR^552^	No NLS predicted
ETS-related transcription factor Elf-1 [87]	*H. sapiens* *M. musculus*	P5: ^177^ GRKTK^181^ ^153^ GRKTK^157^ ^140^ GRKTK^144^ ^118^ GRKTK^122^	^171^QRKRKKGRKTKPPRP^185^
PDZ domain-containing protein 2 [88]	*H. sapiens* *M. musculus* *D. rerio*	P2P3: ^230^GKRTR^234^ ^213^GKRTR^217^ ^48^GKRTR^52^ ^39^GKRTR^43^	^99^KRRGGKKRK^107^ ^210^AKKGKRTRKFGVISR^224^
Nucleolin [89]	*H. sapiens* *M. musculus* *D. rerio*	P2P5: ^739^GKKTK^743^ ^735^GKKTK^739^ ^704^GKKTK^708^ ^701^GKKTK^705^ ^685^GKKTK^689^	^277^AAPGKRKKEMTKQKEAPEAKK^297^ ^382^KPKGRDSKKVR^392^ ^644^PKGEGGFGGRGGGRGGFGGRGGGRGGRGGFGGRGRGGFGGRGGFRGGRGGGGDFKPQGKKTK^705^
DnaJ homolog subfamily C member 21 (DNJC21) [90]	*H. sapiens* *M. musculus*	P2P5: ^604^GKKTK^608^ ^546^GKKTK^550^ ^517^GKKTK^521^ ^504^GKKTK^508^ ^459^GKKTK^463^ ^472^GKKTK^476^ ^334^GKKTK^338^	^181^KRAMEKENKKIRDRARKEKNELVRQLVAFIRKRDKRVQAHRKLV^224^ ^230^EKARKAE^236^ ^380^QKLSKKQKKKKQKS^393^ ^452^KSVPKSKGKKTKDVKKSVK^470^ ^523^NKKEKRRSR^531^
Chromo-domain helicase DNA-binding protein 6 (CHD6) [91,92]	*H. sapiens* *M. musculus*	P2P5: ^1187^GKKTK^1191^ ^1186^GKKTK^1190^ ^1185^GKKTK^1189^ ^1184^GKKTK^1188^ ^1164^GKKTK^1168^ ^1163^GKKTK^1167^ ^1139^GKKTK^1143^ ^852^GKKTK ^856^ ^509^GKKTK^513^	^175^GSRTKSKKASREQGPTPVERKKKGKRK^201^ ^236^RSGRQVKR^243^ ^1181^RGRKGKK^1187^ ^2284^RRRRGRRK^2291^ ^2437^GPRRRGRRPR^2446^ ^2652^KRKKKKTK^2659^
Lupus La protein [93]	*H. sapiens* *D. rerio*	P2P5: ^359^GKKTK^363^ ^357^GKKTK^361^	^328^KWKSKGRRFKGKGKGNKAAQPGSGKGKV^355^
Brefeldin A-inhibited guanine nucleotide-exchange protein 1 [89]	*H. sapiens* *M. musculus* *D. rerio*	P2P5: ^4^GKKTK^8^	No NLS predicted
SWI/SNF-related matrix-associated actin-dependent regulator of chromatin subfamily B member 1 [94]	*H. sapiens* *M. musculus*	P2P5: ^69^GKKTK^73^	No NLS predicted
Lysine-specific demethylase PHF2 [95]	*H. sapiens* *M. musculus*	P2P3P5: ^1069^GKRTK^1073^ ^1068^GKRTK^1072^ ^1034^GKRTK^1038^	^63^KKKR^66^, ^888^KKR^890^, ^942^KNRKKKNTKRKP^953^ ^487^KVSKKKTSKTVKMPKPSKIPKPPKSPKPPKTLKLKDGSKKKGKK^530^ ^827^RKIGGGNKGTGKRLLKR^843^ ^1066^AKGKRTKKGMATAKQRLGKILKIHRN^1091^
Thymocyte nuclear protein 1 [46]	*H. sapiens* *M. musculus*	P2P3P5: ^44^GKRTK^48^ ^21^GKRTK^25^ ^20^GKRTK^24^	^26^RPRKRQTGTAGPDRKKLSGKR^46^

^1^ All listed proteins are known to function in the nucleus of infected cells. ^2^ NLSs were predicted using NLStradamus [48]. Overlapping regions of the predicted NLS containing GRKTR or its variants are highlighted in red.

**Table 5:** Alignments of several NLSs indicating their binding to importin-α deduced from the crystal structure of importin-α in complex with the NLS.

NLS Type	Minor Binding Site P_1′_ P_2′_ P_3′_ P_4′_ P_5′_	Linker	Major Binding Site P_1_ P_2_ P_3_ P_4_ P_5_	PDB id
SV40 large T-antigen	K **K** R K		K **K** K R K	1EJL/1BK6 ^1^
hPLSCR1-NLS			G **K** I S K	1Y2A
hPLSCR4-NLS	I **R** KW N			3Q5U
Guα -NLS	K **R** S F			3ZIN
A89-NLS	K **R** K Y W			4B8P ^2^
B54-NLS	K **R** K R H			2YNS ^2^
TPX2	K **R** K H		V **K** M I K	3KND
C-Myc	K **R** V K L		A **K** R V K	1EE4 ^1^
Nucleoplasmin	K **R** P A A	TKKAG	K **K** K K L	1EJY/1EE5 ^1^
Kap60-IBB	R **R** R R D	TQQVELRKAKRDEA	A **K** R R N	1WA5 ^1^
h1NLS	K **R** K D P	DSDDWSES	S **K** E N K	4XZR ^1^
h2NLS	K **R** K R E	QISTDNEAKMQIQEEKS	K **K** K R K	4PVZ ^1^
hRCC1	K **R** R S	PPADAIP	S **K** K V K	5TBK
yRCC1	K **R** T V A	TNGDASGAH	K **K** M S K	5T94 ^1^
BFDV Cap NLS			Y R **R** R R R Y	4HTV
Influenza A NP-NLS1	K **R** S Y E			4ZDU
WT NLS2	R **K** T R		G **R** K T R	5V5O
NLS2 P3 variant	R **R** T R		G **R** R T R	5V5P

^1^ Denotes yeast importin-α (Kap60). ^2^ Denotes rice importin-α. In all other cases, mammalian importin-α was co-crystallized with NLSs. Basic residues at P_2_ (major binding site) or P_2’_ (minor binding site) are indicated in bold.

## Data Availability

Not applicable.

## Supplementary Materials

The following supporting information can be downloaded at: https://www.mdpi.com/article/10.3390/cells11192957/s1, Figure S1: functional analysis of the three NLS2 variants absent in influenza A virus NP; Table S1: NLS2 variants located at NP amino acid residues different than 212–216; Table S2: BLASTP E-values for influenza A NP and proteins from DNA virus families reported in Figure 2 and Figure 4; Table S3: proteins from DNA virus families containing variants of the sequence GRKTR; Table S4: nuclear proteins containing variants of the sequence GRKTR found only in humans and their predicted NLSs; Table S5: nuclear proteins from different cellular organisms containing the sequence GRKTR or its variants and their predicted NLSs; Table S6: BLASTP E-values for nuclear proteins from different cellular organisms reported in Figure 5.
