# Supplementary material for: Bioinformatics and Functional Analysis of a New Nuclear Localization Sequence of the Influenza A Virus Nucleoprotein
Source: Cells. 2022 Sep 22;11(19):2957. doi: 10.3390/cells11192957 (PMC9563117; doi:10.3390/cells11192957)
Supplement: Supplementary file 1 [file cells-11-02957-s001.zip › cells-1896441-supplementary.pdf]

cells MDPI

Supplementary Figures and Tables

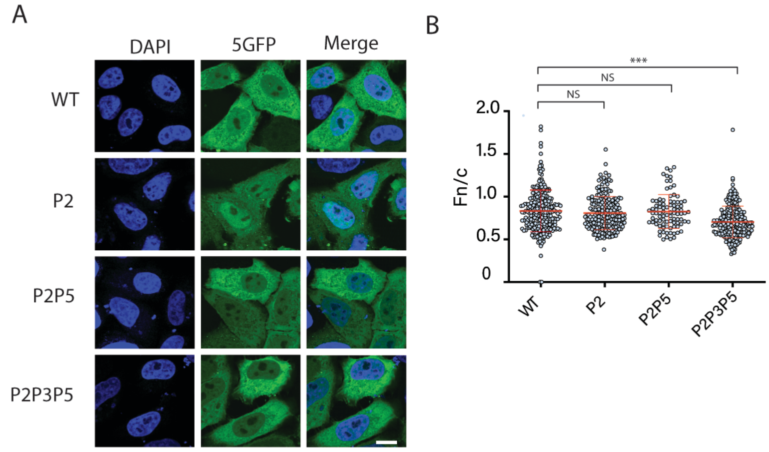

Figure S1. Functional analysis of the three NLS2 variants absent in influenza A virus NP. (**A**) Confocal images of HeLa cells transfected with plasmids expressing 5GFP or three remaining NLS2 variants fused to 5GFP 24 hours post-transfection. Nuclei were stained with DAPI. Scale bar, 10 µm. (**B**) Quantification of the ratio of nuclear to cytoplasmic fluorescence (Fn/c) from the experimental conditions shown in B. Shown is the means ± standard error of the means scored from 85-100 cells for each condition from three independent experiments. (NS, not significant; ***$p < 0.001$, one-way ANOVA followed by Tukey's tests).

Table S1. NLS2 variants located at NP amino acid residues different than 212-216.

NLS2 Variant	Location of NLS2 in the Protein	Accession Number
WT	218 to 222	BAL70445.1, BAL70455.1, BAK41897.1
P3	213 to 217	ADU16942.1, ADU53204.1
P3	218 to 222	ACU51033.1, ACU51030.1, BAM78353.1, ATW75159.1, ABY81789.2, ANZ03693.1, ANK78256.1, BAM78385.1, ATW75162.1, AAN46830.1, AAV30838.1, 2Q06_A, 2Q06_B, ATW75160.1, ABY70960.1, BAK41893.1, BAK41894.1, BAK41895.1, BAK41896.1, BAK41898.1, ATW75163.1, ATW75164.1, ATW75165.1, ATW75166.1, ABF48471.1, BAM65747.1, ATW75168.1, BAM78369.1, ACU51029.1, ACU51031.1, ATW75155.1, ATW75156.1, ATW75158.1, BAL70427.1, ADI52818.1, ADI52820.1, BAL70465.1, AEX30601.1, ATW75169.1, ATW75157.1, BAG49623.1, ALT19014.1, ACR01026.1, ATW75167.1, BAM78401.1, AAV30830.1, ACU51032.1, ACU51034.1, ACU51035.1, APP91838.1

Table S2. BLASTP E-values for influenza A NP and proteins from DNA virus families reported in Figures 2 and 4 [1].

Variant	Influenza A NP	*Adenoviridae*	*Herpesviridae*	*Circoviridae*	*Hepadnaviridae*
WT	224.0 ± 0.0	No hit	No hit	No hit	No hit
P2	No hit	13.0 ± 0.0	71.0 ± 0.0	No hit	No hit
P3	158.0 ± 0.0	6.7 ± 0.1	35.0 ± 0.0	2.2	No hit
P5	318.0 ± 0.0	No hit	71.0 ± 0.0	No hit	No hit
P2P3	224.0 ± 0.0	9.2 ± 0.0	50.0 ± 0.0	No hit	No hit
P2P5	No hit	No hit	Not hit	No hit	No hit
P3P5	224.1 ± 0.0	9.2 ± 0.0	No hit	No hit	No hit
P2P3P5	No hit	No hit	No hit	No hit	78.0 ± 0.0
Random peptide (GLVTV)	No hit	No hit	No hit	6.9 ± 0.0	No hit
SV40 Large T-antigen NLS	No hit	No hit	No hit	No hit	No hit

[1] Mean of E-value ± standard error of the mean are listed.

Table S3. Proteins from DNA virus families containing variants of the sequence GRKTR.

GRKTR Variant	Protein	Accession Number
		Adenoviridae family
P2	Minor core protein pV	AQQ73631
	Viral RNA splicing factor L4-33 kDa	YP_009373251, ART33376
	Viral transcription factor L4-22 kDa	YP_002822221, ACJ14521, YP_002822220, ACJ14522
P3	Precursor terminal protein pTP	YP_007518314, AEK98448, QHR77492
	Encapsidation protein L1-52/55 kDa	QJC19249
	Late L2 mu core protein pX	AWB14650, YP_004123745, ADR77841, AP_000057, AAB05441, P68974, P68975
P2P3	Minor core protein pV	AJE59778, AGT76023, AJE59852, AKQ98460, AJE59724, AJE59783, AJE59788, AJE59808, AKQ98454, AFQ34581, AJE59813, AFQ34346, AGT76107, AGT77115, AFQ34307, AJE59734, AJE59798, AJE59719, AJE59754, AJE59759, AJE59769, AKQ98442, AJE59803, AGT77293, AJE59709, AJE59764, AGT76151, AGT75892, AGT76065, AGT76536, AJE59857, AJE59862, AKQ98376, AAZ13829, AFQ34385, AGT75547 AGT75675, AUN87399, AFQ34424, AJE59867, AJE59739, AJE59744, AAZ15253, AJE59881, AJE59895, AKQ98448, YP_002213776, AJE59876, AKQ98418, AET87309, AAW33441, AAW33258, ANQ44536, AAW33166, ABB1780, QEQ50111, QEQ50148, AMB61156, AP_000545, AAR89960, AAT97584, QBG39296, QEQ50074, AMB61089, QID75502, AAT97543, ACX32405, AEC11867, AAW33399, AMB61124, AYI99658, AET87268, AET87227, AET87145, AET87186, QFR07974
P3P5	Hexon-associated structural protein (pVIII precursor)	AAC40820
		Herpesviridae family
WT	Tegument protein UL37	YP_00917690, AIU39240, AKV40709, AIU39350, AKI81626, AKI81730, AAY59063
P2	GP84	CDI95420, YP_007417850, AGE11554, BAJ78542
	DNA polymerase processivity subunit	ACY41129, NP_044897, AAB66449, CAA70274
P3	Tegument protein VP22	BBM13221
P5	Tegument protein UL32	AEQ32128
P2P3	Assembly protein M80	CCE57080, AWV68505, ADD10452, CCE56751, CCE56914, CCE57244, YP_214083, AAA46000, AQQ81427, AWV68681, CCE56585, CCE57408, AAA46001, CAP08120, ACE95421, ACE95257, ACE95585
		Circoviridae family
P3	Capsid protein	QFR58250
		Hepadnaviridae family
P2P5	X protein	AGO96886

Table S4. Nuclear proteins containing variants of the sequence GRKTR found only in humans and their predicted NLSs.

Protein [1]	Organism	Putative NLS2 Variant	Predicted NLS [2]
Ubl carboxyl-terminal hydrolase 18	*H. sapiens*	P2:<br>^{234}GKKTR^{238}<br>^{160}GKKTR^{164}	No NLS predicted
Transcriptional repressor CTCFL	*H. sapiens*	P3:<br>^{581}GRRTR^{585}<br>^{531}GRRTR^{535}<br>^{376}GRRTR^{380}<br>^{319}GRRTR^{323}	^{576}AASGKGRRTRKRKQTILKEATKGQKEA^{602}
Sororin	*H. sapiens*	P3:<br>^{3}GRRTR^{7}	^{12}AQRSGPRAPSPTKPLRRSQRK^{32}<br>^{212}PEKQKRKKKKMPEILSLRGRQRA^{234}
N-lysine methyltransferase SETD6	*H. sapiens*	P3:<br>^{46}GRRTR^{50}	No NLS predicted
Probable rRNA-processing protein EBP2	*H. sapiens*	P2P3:<br>^{349}GKRTR^{353}<br>^{294}GKRTR^{298}	^{277}QRKKAGAKGQQMRKGPSAKRRYKNQKFGF GGKKKGSKW^{314}<br>^{328}AKTAHGRGLKRPGKKGSNKRPGKRTREKMK NRTH^{361}
Torsin-1A-interacting protein 1	*H. sapiens*	P2P3:<br>^{82}GKRTR^{86}	No NLS predicted
Interferon-induced, double-stranded RNA-activated protein kinase]	*H. sapiens*	P2P3:<br>^{443}GKRTR^{447}<br>^{402}GKRTR^{406}	No NLS predicted
Transforming growth factor beta regulator 1	*H. sapiens*	P2P5:<br>^{133}GKKTK^{137}	^{19}KARMKKLPKKSQNEKYRLKYLRLRKA^{44}<br>^{133}GKKTKKEKKEKGKENNKLEVLKKTCKKKKM AGGARK^{168}
Histone-lysine N-methyltransferase NSD2	*H. sapiens*	P2P5:<br>^{1218}GKKTK^{1222}<br>^{1149}GKKTK^{1153}<br>^{437}GKKTK^{441}	^{164}KARRNRKRS^{172}<br>^{1217}KGKKTKKKTRRRRAKGEGKRQ^{1237}<br>^{1339}EKPPPEPGKPKGKRRRRRGWRRVTEGK^{1365}
Chromobox protein homolog 5	*H. sapiens*	P2P5:<br>^{2}GKKTK^{6}	^{69}KYKKMKEGENNKPREKSEGNKRKS^{92}<br>^{103}SKKK^{106}
ATP-binding cassette sub-family F member 1	*H. sapiens*	P2P5:<br>^{123}GKKTK^{127}	^{28}VKKGKKDKKIKK^{39}<br>^{74}KKKRDTRKGRRKK^{86}<br>^{119}KPRGGKK^{125}<br>^{211}KEPPKQGKEKAKK^{223}<br>^{518}KKLKELKAGGKSTK^{531}
Cyclin-F	*H. sapiens*	P3P5:<br>^{567}GRRTK^{571}<br>^{259}GRRTK^{263}	^{21}RRIRRR^{26}

[1] All listed proteins are known to function in the cell nucleus.
[2] NLSs were predicted using NLStradamus (Nguyen et al. 2009; BMC Bioinformatics. 10:202). Overlapping regions of the predicted NLSs containing GRKTR or its variants are highlighted in red.

Table S5. Nuclear proteins from different cellular organisms containing the sequence GRKTR or its variants and their predicted NLSs.

Protein.[1]	Species	Putative NLS2 Variant	Predicted NLS.[2]
Suppressor of mec-8 and unc-52 protein	*G. max*	WT:<br>^{513}GRKTR^{517}<br>^{515}GRKTR^{517}	^{6}KKNPKEKPIRRKEEKPEEPEVPKYRDRAKER^{36}<br>^{215}GKILKKKRKEKDAKGK^{230}
Spliceosomal protein Beag	*D. melanogaster*	WT:<br>^{504}GRKTR^{508}	^{77}LRRKKK^{82}<br>^{314}GGRNKKNKKRDKDK^{327}
Methyl-CPG-binding domain protein 13	*A. thaliana*	WT:<br>^{54}GRKTR^{58}	No NLS predicted
U-box domain-containing protein	*G. max*	WT:<br>^{509}GRKTR^{513}<br>^{501}GRKTR^{505}	No NLS predicted
Regulator of apoptosis protein XIAP-associated factor	*M. musculus*	WT:<br>^{130}GRKTR^{134}	No NLS predicted
Atpase family AAA domain-containing protein 5	*M. musculus*	P2:<br>^{477}GKKTR^{481}	^{111}KRKRK^{115}<br>^{202}RKRKR^{206}<br>^{466}PKEKSKKPNKKGKKTR^{481}<br>^{654}PKKSKKSSKK^{663}<br>^{1543}LRKSQKRKQKK^{1553}
Transcription factor Scarecrow-like protein 14	*G. max*	P2:<br>^{289}GKKTR^{293}	^{290}KKTRSKKGS^{298}
Origin of replication complex subunit 1	*G. max*	P2:<br>^{579}GKKTR^{583}<br>^{576}GKKTR^{580}	^{106}KKK^{108}<br>^{132}RKREGEGGVVTRAKRRKSENREKSAKLP^{159}
Lysine-specific demethylase 6B	*M. musculus*	P2:<br>^{1069}GKKTR^{1073}	^{972}GSGKRRQKEHRRHRRACRDSVGRRPREGRAKAKAKAPKEKSRR^{1014}
Kinase regulator Mob2	*D. melanogaster*	P2:<br>^{394}GKKTR^{398}<br>^{232}GKKTR^{236}<br>^{203}GKKTR^{207}<br>^{97}GKKTR^{101}	^{972}GSGKRRQKEHRRHRRACRDSVGRRPREGRAKAKAKAPKEKSRR^{1014}
Ethylene-responsive transcription factor 9	*G. max*<br>*A. thaliana*	P2:<br>^{46}GKKTR^{50}<br>^{45}GKKTR^{49}<br>^{43}GKKTR^{47}<br>^{42}GKKTR^{45}	No NLS predicted
SMAD/FHA domain-containing transcription factor	*A. thaliana*	P2:<br>^{183}GKKTR^{187}	No NLS predicted
Smg-4/UPF3 family protein	*A. thaliana*	P3:<br>^{185}GRRTR^{189}	^{182}RRGGRRTRVVSANKPSPRPSKRNSEKKKYVEK^{213}
Lupus La protein homolog	*M. musculus*	P3:<br>^{374}GRRTR^{378}<br>^{373}GRRTR^{377}	^{199}SKVEAKLKAKQ^{209}<br>^{329}KSKGGHAGGRFKGSHVFTAARRFKGKGKGNRPGYAGAPKGRGQFHGRRTRFDDDDRRRGPMKRGRDGRDREEPASKHKKRENGARDK^{415}
Recq-mediated genome instability protein 1	*G. max*	P3:<br>^{263}GRRTR^{267}	^{255}LKKPPRPRGRRTR^{267}
E3 ubiquitin-protein ligase RNF169	*D. rerio*	P3:<br>^{190}GRRTR^{194}	^{526}RRGKKRSQK^{534}
Inflammatory response factor strawberry notch homolog	*C. elegans*	P2:<br>^{1789}GRRTR^{1793}	^{1253}KDRKAKKRKRDEEEAERLREKVRKREERREKKRRRAIRRAEREKQRR^{1299}

Protein timeless homolog	*C. elegans*	P2:<br>^{173}GRRTR^{177}	^{307}RKAKKRAPRNRR^{318}<br>^{520}LKKVTKKVKVRKATKKSK^{537}<br>^{1011}RSRKKVA^{1017}
Myb domain-containing protein		P2:<br>^{699}GRRTR^{703}	^{283}DRERDRDRERERERERDRERERERGRDR^{310}<br>^{556}SRERSRSHSRSRSRSRSRSHSR^{577}<br>^{621}KRKSGGDDGKDVKRRKTGS^{639}
Nuclear inhibitor of protein phosphatase 1	*D. melanogaster*	P2:<br>^{186}GRRTR^{190}	No NLS predicted
Protein MAIN-LIKE 2-like	*G. max*	P2:<br>^{23}GRRTR^{27}	No NLS predicted
Nuclear receptor corepressor 2	*D. rerio*	P5:<br>^{2268}GRKTK^{2272}<br>^{2258}GRKTK^{2262}<br>^{2252}GRKTK^{2256}<br>^{2242}GRKTK^{2246}<br>^{2230}GRKTK^{2234}<br>^{2204}GRKTK^{2208}<br>^{1646}GRKTK^{1650}	^{653}KIKSEKERKARRKGKA^{668}<br>^{804}KKEVKKKEH^{812}<br>^{1674}RERERERERERERQRERERERERERERDRERDRDREREKDRDRDRER ^{1717}<br>^{2260}KSKGGRSNGRKTKS^{2273}
Histone h1t	*M. musculus*	P5:<br>^{165}GRKTK^{169}	^{0}KPSSKRRGKKPGLAPARKPRGF^{41}<br>^{110}LSKKAASGNDKGKGKKSASAKAKKMGLPRASRSPKSSKTKAVKKPKATPTKASGSGRKTKGAKGVQQRK-SPAKARAANPNSGKAKMVMQKTDLRKAAGRK^{209}
Winged-helix DNA-binding transcription factor family protein	*A. thaliana*	P5:<br>^{35}GRKTK^{39}	^{29}KKPAAKGRKTKNVKEVKEKKTVAAAPKKRT^{58}
AGAMOUS-like 57	*A. thaliana*	P5:<br>^{9}GRKTK^{13}	^{7}AKGRKTKGKQKI^{18}
MRX complex nuclease subunit	*S. cerevisiae*	P5:<br>^{650}GRKTK^{654}	^{588}KPKRVRTATKKKI^{600}<br>^{651}RKTKTKTRPAASTKTASRRGKGRASRTPKTDILGSLLAKKRK^{692}
Transcription factor MIG1	*S. cerevisiae*	P5:<br>^{159}GRKTK^{163}	^{87}RRIHTNSHPRGKRGRKKK^{104}
Acyl-CoA N-acyltransferase with RING/FYVE/PHD-type zinc finger protein	*A. thaliana*	P5:<br>^{484}GRKTK^{488}	^{64}RRSGVDRKTLGAKEKFVRKR^{83}<br>^{266}RKSKRKKSEYSKPKKE^{281}<br>^{302}RRGRRGGGTDKQRL^{315}<br>^{396}RKAKKTR^{402}
Topoisomerase 3alpha	*A. thaliana*	P5:<br>^{584}GRKTK^{588}	^{862}GRRGSRGRGRGGRGGQSSGGR^{882}
Lysine-specific demethylase 4C	*M. musculus*	P5:<br>^{694}GRKTK^{698}<br>^{677}GRKTK^{681}<br>^{602}GRKTK^{606}<br>^{496}GRKTK^{500}	^{379}RKKKLRKPPKSLQGNK^{394}
Probable serine/threonine-protein kinase At1g54610	*G. max*	P5:<br>^{523}GRKTK^{527}	^{423}GAKKVRVRERERG^{435}
Caspase-14	*M. musculus*	P5:<br>^{151}GRKTK^{155}	No NLS predicted
ELM2 and Myb/SANT-like domain containing 1a	*D. rerio*	P5:<br>^{913}GRKTK^{917}	No NLS predicted
Mitotic deacetylase associated SANT domain protein a	*D. rerio*	P5:<br>^{873}GRKTK^{877}	No NLS predicted
Trithorax	*D. melanogaster*	P5:<br>^{1224}GRKTK^{1228}<br>^{856}GRKTK^{860}	No NLS predicted
NAC domain-containing protein 90	*G. max*<br>*A. thaliana*	P5:<br>^{123}GRKTK^{127}<br>^{125}GRKTK^{129}	No NLS predicted

Protein Wiz	*D. rerio*	P2P3:<br>^{275}GKRTR^{279}<br>^{269}GKRTR^{273}	^{252}RRKKR^{256}<br>^{276}KRTRGRPPGKGRNKT^{290}<br>^{598}RRKDVAFKSIGNKRGRGGRGRRRG^{621}
Tetratricopeptide repeat protein 27	*M. musculus*	P2P3:<br>^{276}GKRTR^{280}	No NLS predicted
Kinesin-like protein KIF22	*D. rerio*	P2P3:<br>^{363}GKRTR^{367}	No NLS predicted
Meiotic recombination protein SPO11-1	*G. max*	P2P3:<br>^{3}GKRTR^{7}	No NLS predicted
Transcription factor DIVARICATA	*G. max*	P2P3:<br>^{74}GKRTR^{78}	No NLS predicted
RNA polymerase III subunit C160	*D. melanogaster*	P2P3:<br>^{1236}GKRTR^{1240}	No NLS predicted
Recq-mediated instability protein (DUF1767)	*A. thaliana*	P2P3:<br>^{282}GKRTR^{286}	No NLS predicted
DNA binding protein	*A. thaliana*	P2P3:<br>^{23}GKRTR^{27}	No NLS predicted
Small subunit processome component 20 homolog	*M. musculus*	P2P5:<br>^{2506}GKKTK^{2510}	^{1321}KKKKN^{1325}<br>^{2505}KGKKTKKK^{2512}<br>^{2727}KRALRKKRKAL^{2737}<br>^{2744}DIAAKKKLKKHKNKSEAKKRKIEFLRPGYKAKRQK^{2778}
Methyl-CPG-binding domain 9	*A. thaliana*	P2P5:<br>^{1779}GKKTK^{1783}	^{734}RKAKKPKL^{741}<br>^{916}RGRK^{919}<br>^{1353}RRK^{1355}<br>^{1776}KKRGKKTK^{1783}<br>^{2162}RVRRSGKKRKEP^{2173}
High mobility group B4	*A. thaliana*	P2P5:<br>^{25}GKKTK^{29}	^{17}LKTRGRKAGKKTKKDPNQPKRP^{38}
FACT complex subunit ssrp1-B	*C. elegans*	P2P5:<br>^{552}GKKTK^{556}	^{525}PKKKESKEKKNKREKKEKPVKEKAVKKGKKTKDPNEPK^{562}
Galactose oxidase/kelch repeat superfamily protein	*A. thaliana*	P2P5:<br>^{2}GKKTK^{6}	^{3}KKTKKPGKGKEKTERKTAKADEKKARREGKKL^{34}<br>^{353}SKDKAK^{358}<br>^{554}KSLRRKEKRARI^{565}<br>^{657}RKGGIAKKKR^{666}
ATPase family AAA domain-containing protein 5	*D. rerio*	P2P5:<br>^{911}GKKTK^{915}	^{93}KPVRGRGQKRTRKTKDKKKSK^{113}<br>^{182}KKDGGNKKSALRRNRKAK^{199}<br>^{443}KKMKVRKSGKKAQAKKA^{459}<br>^{595}AKKRKQAKKLVQKAKALQQNKKDTKKEKETVRRS^{629}<br>^{897}KRK^{899}<br>^{941}GRGRRGRSLRQK^{952}<br>^{1151}PRRVVSSPRKPPQSPR^{1166}
Cat eye syndrome critical region protein 2 homolog	*M. musculus*	P2P5:<br>^{479}GKKTK^{383}<br>^{407}GKKTK^{411}<br>^{266}GKKTK^{270}	^{195}GKTGKRRGRPPKRKK^{209}<br>^{358}KREMEEKVKAVEDRAKRRKLR^{378}<br>^{537}KREKRRSRSGR^{547}
Selenocysteine insertion sequence-binding protein 2-like	*M. musculus*	P2P5:<br>^{480}GKKTK^{484}<br>^{369}GKKTK^{373}<br>^{157}GKKTK^{161}	^{133}KGRRRR^{138}<br>^{333}KSQKK^{337}<br>^{444}KAKKGKEKEIAKLKRPTALKKVILKEREEKKGR^{476}
E3 ubiquitin-protein ligase UHRF2	*M. musculus*	P2P5:<br>^{648}GKKTK^{652}<br>^{612}GKKTK^{616}<br>^{424}GKKTK^{428}<br>^{388}GKKTK^{392}	^{408}KLSKKKAK^{415}

Nucleolar protein 56	*G. max*	P2P5: ^{448}GKKTK^{452}	^{449}KKTKKKKQK^{457}<br>^{479}KSEKKKKKEKRK^{490}<br>^{518}KKKKNKKDSNGEALEAGGESKKKKKKSKKK^{547}
Protein DEK	*G. max*	P2P5: ^{183}GKKTK^{187} ^{167}GKKTK^{171}	^{91}KEKEKAKGKGKRRRSKKDPVTPAPSERPTRERK^{123}<br>^{272}KGKKRTRKSFPSKS^{285}
SAGA histone acetyltransferase complex subunit SPT7	*S. cerevisiae*	P2P5: ^{1064}GKKTK^{1068}	^{333}KGKKKRS^{339}
E3 ubiquitin-protein ligase TRIM69	*M. musculus*	P2P5: ^{386}GKKTK^{390} ^{368}GKKTK^{372}	No NLS predicted
PAZ domain-containing protein / piwi domain-containing protein	*A. thaliana*	P2P5: ^{166}GKKTK^{170}	No NLS predicted
AGAMOUS-like-56	*A. thaliana*	P2P5: ^{3}GKKTK^{7}	No NLS predicted
NAC (No Apical Meristem) domain transcriptional regulator superfamily protein	*A. thaliana*	P2P5: ^{157}GKKTK^{161}	No NLS predicted
CDKN2A-interacting protein	*D. rerio*	P2P5: ^{130}GKKTK^{134} ^{129}GKKTK^{133} ^{43}GKKTK^{47}	No NLS predicted
Fap1p	*S. cerevisiae*	P2P5: ^{559}GKKTK^{563}	No NLS predicted
Serum response factor homolog	*C. elegans*	P2P5: ^{41}GKKTK^{45}	No NLS predicted
IQ domain-containing protein IQM2	*G. max*	P2P5: ^{325}GKKTK^{329}	No NLS predicted
Probable WRKY transcription factor 32	*G. max*	P2P5: ^{315}GKKTK^{319}	No NLS predicted
PR domain zinc finger protein 10	*D. rerio*	P3P5: ^{425}GRRTK^{429}	^{405}RPKSRGRGRGRKRFGGARRPGRRT^{428}<br>^{711}RKDKLREHMQRMHNPEREAKKADRIHRTKA^{740}
Bromodomain testis-specific protein	*D. rerio*	P3P5: ^{264}GRRTK^{268}	^{455}KPKKKEKSKKKKKKERESSKRKGEEMKKPAKILKRSSSSKSSGRK^{499}<br>^{581}RKKTKETNKNKPPKKSKIKEKD^{602}<br>^{666}TKRKQSKGPGHANKIKKKAL^{685}
Xeroderma pigmentosum, complementation group C	*D. melanogaster*	P3P5: ^{1043}GRRTK^{1047} ^{1042}GRRTK^{1046}	^{61}KRGSDHKAPSGIKGSSVKKRKPTGQS^{86}<br>^{269}KKAGRG^{274}<br>^{295}RIK^{297}<br>^{542}KPKKDKKAGKPAEKESSKSTISKEAEKKNNAKKAEAKPLSK^{582}
Transcription factor unc-3	*C. elegans*	P3P5: ^{244}GRRTK^{248}	No NLS predicted
Pre-mRNA processing factor 31	*D. discoideum*	P2P3P5: ^{330}GKRTK^{334}	^{327}PEEGKRTKRGGKKARL^{342}
Crossover junction endonuclease MUS81	*G. max*	P2P3P5: ^{93}GKRTK^{93}	^{92}KGKRTK^{97}
Cyclin-D3-1-like	*G. max*	P3P5: ^{311}GRRTK^{315}	^{313}RTKLKKR^{319}
Toutatis	*D. melanogaster*	P2P3P5: ^{2611}GKRTK^{2615} ^{2574}GKRTK^{2578} ^{2538}GKRTK^{2542}	^{1088}LARNKEKARQEKNSKLEQQRKDKE^{1111}<br>^{2511}KNKSSKKKSSAKKQATPSKKQQQKKNKKE^{2539}<br>^{2612}KRTKKKSGGKRRR^{2624}<br>^{2746}PPPKKRSAGGTSGSSSKSRRDRDRESGGSAKRR^{2778}<br>^{3011}LKQEKKEKHATK^{3022}
Restriction endonuclease, type	*A. thaliana*	P2P3P5:	^{339}KSKEHVKNKSGKKRNS^{354}

II-like superfamily protein		^{267}GKRTK^{271}	
Fanconi anemia group E protein	*M. musculus*	P2P3P5:<br>^{193}GKRTK^{197}	No NLS predicted
Protein POLLENLESS 3-LIKE 2	*G. max*	P2P3P5:<br>^{155}GKRTK^{159}	No NLS predicted
Transcription factor bhlh91	*G. max*	P2P3P5:<br>^{193}GKRTK^{197}<br>^{180}GKRTK^{184}	No NLS predicted
Proliferation marker protein Ki-67	*D. rerio*<br>*M. musculus*	P5:<br>^{1807}GRKTK^{1811}<br>^{1636}GRKTK^{1640}<br><br>P2P5:<br>^{2215}GKKTK^{2219}<br>^{2071}GKKTK^{2075}<br>^{1895}GKKTK^{1899}<br>^{1388}GKKTK^{1392}<br>^{1244}GKKTK^{1248}<br>^{1147}GKKTK^{1151}<br>^{1124}GKKTK^{1128}<br>^{1068}GKKTK^{1072}<br><br>P2P3P5:<br>^{2215}GKRTK^{2219}<br>^{1388}GKRTK^{1392}<br>^{1147}GKRTK^{1151}<br>^{1003}GKRTK^{1007}<br>^{827}GKRTK^{831}	^{558}GRK^{560}<br>^{627}KRQRRP^{632}<br>^{1311}RRPR^{1314}<br>^{1526}RKPAKR^{1531}<br>^{1900}RRPR^{1903}<br>^{2137}KRRPR^{2141}<br>—<br>^{2725}RPRRV^{2729}<br>^{2965}PVPEKKRAASSKRH^{2978}
C2H2-like zinc finger protein	*A. thaliana*<br>*G. max*	P2P5:<br>^{265}GKKTK^{269}<br><br>P2P3P5:<br>^{24}GKRTK^{28}<br>^{20}GKRTK^{24}<br>^{18}GKRTK^{22}<br>^{17}GKRTK^{21}	No NLS predicted

[1] All listed proteins are known to function in the cell nucleus

[2] NLSs were predicted using NLStradamus (Nguyen et al. 2009; BMC Bioinformatics. 10:202). Overlapping regions of the predicted NLSs containing GRKTR or its variants are highlighted in red.

Table S6. BLASTP E-values for nuclear proteins from different cellular organisms reported in Figure 5.[1]

Variant	*M. musculus*	*H. sapiens*	*D. rerio*	*G. max*	*A. thaliana*	*D. melano-gaster*	*C. elegans*	*S. cerevisiae*
WT	341.2 ± 0.1 ($p < 0.001$)	342.6 ± 0.6 ($p < 0.001$)	343.0	343.0 ± 0.0 ($p < 0.001$)	343.0 ± 0.0 ($p < 0.001$)	343.0	No hit	No hit
P2	485.5 ± 0.2 ($p < 0.001$)	489.5 ± 0.3 ($p < 0.001$)	488.25 ± 0.8 ($p < 0.001$)	490.0 ± 1.3 ($p < 0.001$)	491.7 ± 0.9 ($p < 0.001$)	487.6 ± 0.43	489.0	489.0
P3	242.2 ± 0.5 ($p < 0.001$)	242.5 ± 0.2 ($p < 0.001$)	242.0 ($p < 0.001$)	242.5 ± 0.5 ($p < 0.001$)	242.0 ($p < 0.001$)	243.0	241.0± 0.0 ($p < 0.001$)	No hit
P5	487.2 ± 0.6 ($p < 0.001$)	487.0 ± 0.0 ($p < 0.001$)	484.3 ± 0.1 ($p < 0.001$)	489.7 ± 0.8 ($p < 0.001$)	488.1 ± 1.4 ($p < 0.001$)	484.0 ± 0.0	No hit	486.5 ± 0.50
P2P3	341.3 ± 0.1 ($p < 0.001$)	342.2 ± 0.3 ($p < 0.001$)	341.3 ± 0.2 ($p < 0.001$)	345.8 ± 1.6 ($p < 0.001$)	344.0 ± 0.3 ($p < 0.001$)	342.0	No hit	No hit
P2P5	691.4 ± 0.9 ($p < 0.001$)	689.9 ± 0.57 ($p < 0.001$)	691.4 ± 1.0 ($p < 0.001$)	690.6 ± 0.2 ($p < 0.001$)	698.3 ± 2.3 ($p < 0.001$)	No hit	692.0 ± 2.0 ($p < 0.001$)	688.5 ± 0.50
P3P5	No hit	343.5 ± 0.5 ($p < 0.001$)	342.0 ± 0.0 ($p < 0.001$)	No hit	No hit	342.0 ± 0.0	344.0 ($p < 0.001$)	No hit
P2P3P5	489.3 ± 1.5 ($p < 0.001$)	No hit	No hit	488.2 ± 0.42 ($p < 0.001$)	487.5 ± 0.7 ($p < 0.001$)	484.0 ± 0.0	No hit	No hit
Random peptide (GLVTV)	751.9 ± 1.8	755.7 ± 2.0	753.2 ± 0.5	752.0 ± 0.0	753.0	No hit	757.4 ± 3.1	No hit
SV40 Large T-antigen NLS	No hit	No hit	No hit	No hit	No hit	No hit	No hit	No hit

[1] Mean of E-values ± standard error of the mean are listed. E-values for each cellular organism were compared with that of the corresponding random peptide by One-way ANOVA followed by Tukey's test using GraphPad Prism (GraphPad Software, Inc., La Jolla, CA).
